# MSCs vs. iPSCs: Potential in therapeutic applications

**DOI:** 10.3389/fcell.2022.1005926

**Published:** 2022-11-02

**Authors:** Kalaiselvaan Thanaskody, Amirah Syamimi Jusop, Gee Jun Tye, Wan Safwani Wan Kamarul Zaman, Sylvia Annabel Dass, Fazlina Nordin

**Affiliations:** ^1^ Centre for Tissue Engineering and Regenerative Medicine (CTERM), Faculty of Medicine, University Kebangsaan Malaysia, Kuala Lumpur, Malaysia; ^2^ Institute for Research in Molecular Medicine (INFORMM), Universiti Sains Malaysia, Gelugor, Malaysia; ^3^ Department of Biomedical Engineering, Faculty of Engineering, Universiti Malaya, Kuala Lumpur, Malaysia; ^4^ Centre for Innovation in Medical Engineering (CIME), Department of Biomedical Engineering, Faculty of Engineering, Universiti Malaya, Kuala Lumpur, Malaysia

**Keywords:** mesenchymal stem cells, induced pluripotent stem cells, therapeutic applications, SARS-CoV-2, COVID-19

## Abstract

Over the past 2 decades, mesenchymal stem cells (MSCs) have attracted a lot of interest as a unique therapeutic approach for a variety of diseases. MSCs are capable of self-renewal and multilineage differentiation capacity, immunomodulatory, and anti-inflammatory properties allowing it to play a role in regenerative medicine. Furthermore, MSCs are low in tumorigenicity and immune privileged, which permits the use of allogeneic MSCs for therapies that eliminate the need to collect MSCs directly from patients. Induced pluripotent stem cells (iPSCs) can be generated from adult cells through gene reprogramming with ectopic expression of specific pluripotency factors. Advancement in iPS technology avoids the destruction of embryos to make pluripotent cells, making it free of ethical concerns. iPSCs can self-renew and develop into a plethora of specialized cells making it a useful resource for regenerative medicine as they may be created from any human source. MSCs have also been used to treat individuals infected with the SARS-CoV-2 virus. MSCs have undergone more clinical trials than iPSCs due to high tumorigenicity, which can trigger oncogenic transformation. In this review, we discussed the overview of mesenchymal stem cells and induced pluripotent stem cells. We briefly present therapeutic approaches and COVID-19-related diseases using MSCs and iPSCs.

## 1 Introduction

Stem cells have the potential to self-renew and can differentiate into a variety of differentiated mature cell types (Weissman, 2000). Embryonic and adult stem cells are both possible sources of stem cells. Their differentiation enables production of differentiated cells that build tissues and organs (Kolios and Moodley, 2013). As a general rule, stem cells possess three main characteristics: First, they are self-renewing, allowing them to reproduce abundantly. Next is clonality, which means cells can be derived from one single cell. Lastly, potency allows cells to differentiate into a variety of cell types (Lin and Talbot, 2014).

**FIGURE 1:**
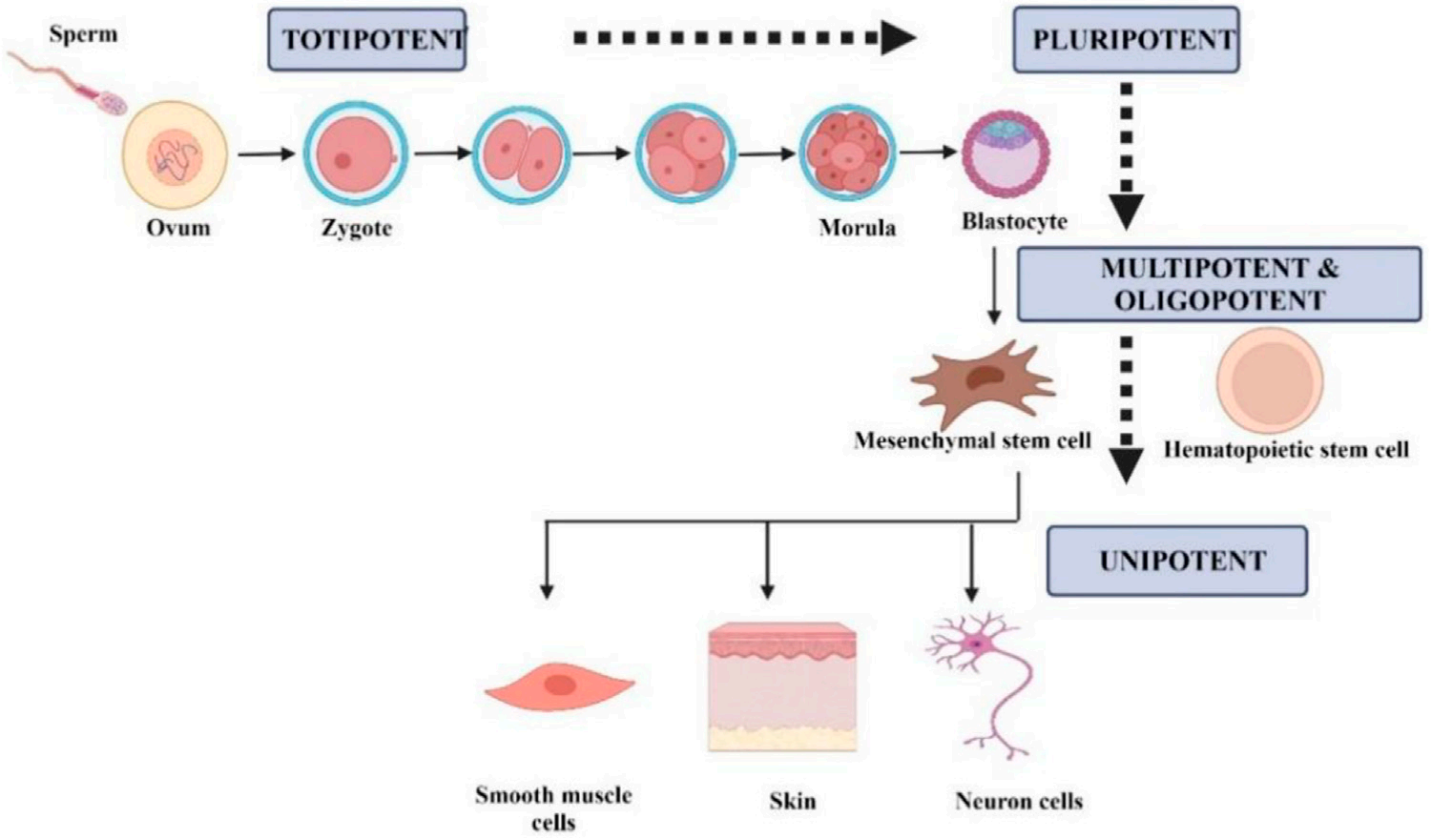
Schematic representation of the differentiation-based stem cells based on differentiation. Created by Biorender.com.

Stem cells can be classified according to their differentiation ability into five groups, namely totipotent, pluripotent, multipotent, oligopotent, and unipotent (Łos et al., 2019). Figure 1 summarizes the classification of stem cells according to the differential potential. Totipotent cell can differentiate into any type of cell and develop into a complete organism. In totipotency, a single cell can produce an entire embryo and its extraembryonic components (Posfai et al., 2021). Pluripotent cells can differentiate into three germ layers, mesoderm, endoderm, and ectoderm, as well as into any type of embryonic and adult cells. Multipotent cells consist of progenitor cells that can differentiate into a limited number of cell types. Oligopotency are cells within a tissue that can differentiate into cells of a specific tissue. Unipotent cells can differentiate into cells of the same type (Loya, 2014; Łos et al., 2019).

Stem cells can be classified according to their origin, such as embryonic stem cells (ESC), adult stem cells, and fetal stem cells (Ilic and Polak, 2011; Kolios and Moodley, 2013). ESC is pluripotent and is derived from the inner cell mass of the blastocyst, which is the preimplantation embryo 5–6 days after fertilization (Kolios and Moodley, 2013). Although these cells can differentiate into three primary germ layers, they can remain undifferentiated in culture for a long time. Blastocytes have two layers of cells, the inner mass of cells that form the embryo and the outer mass of cells that form the placenta called the trophoblast (Ilic and Polak, 2011). To develop the ESC line, cells obtained from the inner cell are separated from the trophoblast and transferred to culture dish under specific conditions.

Adult stem cells, also known as tissue-specific stem cells, are multipotent cells that are responsible for replacing dead cells in the body. These cells are present in many adult tissues such as bone marrow, gastrointestinal tract, the brain, skin, and nerve cells. They are usually tissue-specific and can only differentiate into cell types where the tissues are located. Adult stem cells have a unique ability to differentiate, so that one type of stem cell can differentiate into another type of tissue under appropriate growth conditions (Loya, 2014).

Fetal stem cells are pluripotent and present in the fetus, which contributes to the early development of all prenatal tissues. They are obtained from fetal tissues or extraembryonic tissues such as umbilical cord blood, amniotic fluid, Wharton’s jelly, and the placenta. These cells have to self-replication and differentiation ability (Loya, 2014).

**FIGURE 2:**
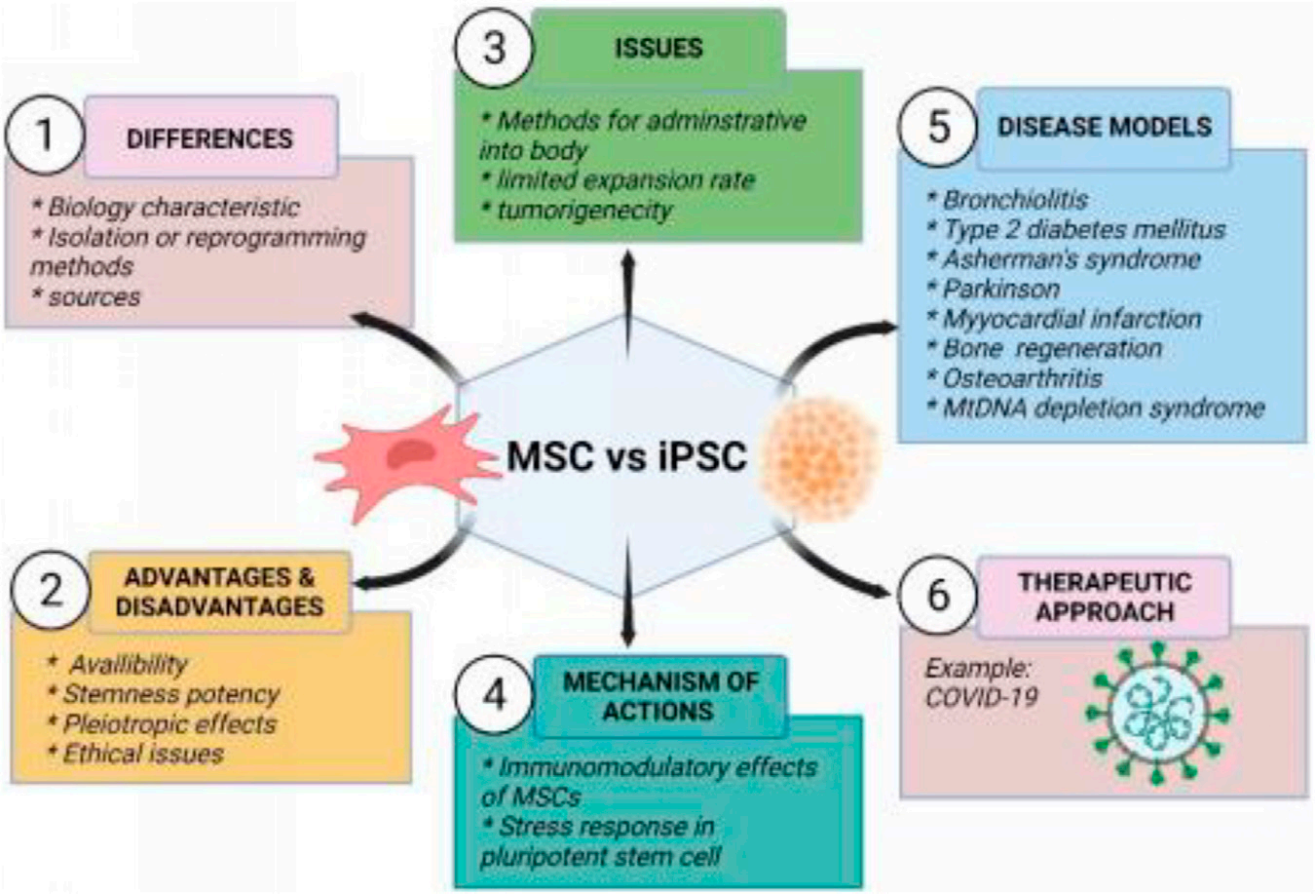
Summary of mesenchymal stem cells and induced pluripotent stem cells. Created by Biorender.com.

The central objective of this review is to highlight the therapeutic potential of MSCs and iPSCs in current clinical studies including COVID-19. In addition, animal models used to study the treatment of certain diseases are discussed in more detail. The characteristics, differences, advantages and disadvantages, main concerned issues, mechanism of action between MSCs and iPSCs, and their therapeutic applications in the disease model are also summarized in Figure 2.

## 2 Differences between MSC and IPSC

### 2.1 Mesenchymal stem cells

**FIGURE 3:**
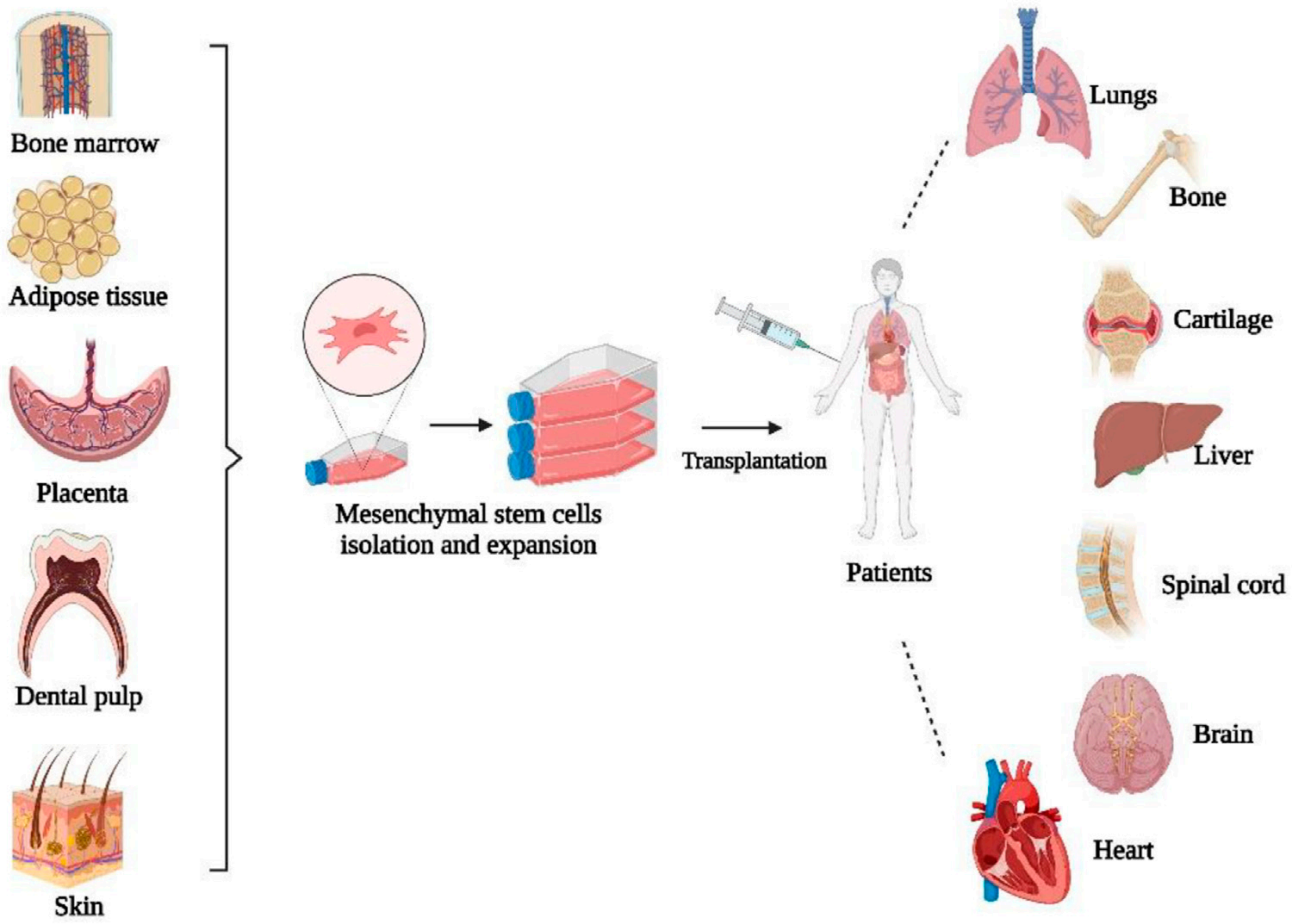
Schematic diagram of MSC-based cell therapy. Created by Biorender.com.

Mesenchymal stem cells (MSCs) are multipotent adult stromal progenitor cells that are heterogeneous, non-hematopoietic, can self-renew and transform into several lineages and cell types (Rastegar et al., 2010). MSCs can be obtained and isolated from a variety of tissues, such as bone marrow, adipose tissue, skin, umbilical cord blood, amniotic fluid, and placenta (Fernandez and Fernandez, 2016). Bone marrow is the main source of MSCs, although MSCs account for only a small proportion of the total number of cells in the bone marrow (Rastegar et al., 2010; Al-Anazi, 2020). MSCs are distinguished by three characteristics, including the ability to differentiate into osteoblasts, adipocytes, and chondrocytes. Second, MSCs should adhere to plastic while maintaining a standard culture condition in a tissue culture flask. The third characteristic is the presence of specific surface markers such as CD105, CD73, and CD90 when measured with flow cytometry (Dominici et al., 2006). Figure 3 shows the schematic diagram of MSC-based cell therapy.

In addition to isolation from tissues, MSCs have been generated by differentiation of ESCs and iPSCs. There are three methods involved in the differentiation of ESCs. An embryoid body method (EB) involves growing embryonic stem cells into three-dimensional structures that can differentiate later into ectodermal, mesodermal, and endodermal cells. However, such lineages are often not uniform since they contain multiple cell lines that are present simultaneously. Another method is to use feeder cells to directly differentiate ESCs into specific lineages. For example, OP9 cells are often used to differentiate ESCs into mesodermal cells such as hematopoietic cells (Hematti, 2011a). Initially, MSCs were derived from ESCs using OP9 cells (Trivedi and Hematti, 2007), but later a method was developed to generate MSCs from human ESCs that do not utilize OP9 or feeder cells (Trivedi and Hematti, 2008). An extracellular matrix (ECM) such as Matrigel, collagen, and gelatin is also used for the two-dimensional differentiation culture method along with cytokines and growth factors that are added to the environment (Hematti, 2011b). OP9 had strong clonogenic ability and could induce osteocytes, chondrocytes, and adipocytes. As OP9 cells were genuine MSCs, such a homogeneous cell line will help define the biological characteristics of MSCs at the stem cell level. The MSC-produced ECM largely replicates the composition and structure, thus mimicking the components of stem cell niche components *in vitro* and maintaining multipotent progenitor cells.

In this study, a one-step method for the generation of from ESC/iPSC derived MSCs was developed, which exhibited typical MSC characteristics as defined by the International Society for Cell Therapy. In culture, these ES-MSCs and iPS-MSCs have normal karyotypes, lack the ability to form teratomas, and show growth and differentiation characteristics similar to those of primary MSCs. This simple EB-free differentiation protocol can be scaled up to allow a wide range of MSC-based therapies due to the ease of harvesting iPSCs and their ability to be grown indefinitely (Y. S. Chen et al., 2012).

In serum-free medium containing the transforming growth factor B pathway inhibitor SB431542, epithelial-like monolayer cells were generated from ESC/iPSC. SB431542 is believed to induce hESC differentiation by inhibiting SMAD2/3 phosphorylation by suppressing activin receptor-like kinase (ALK) receptors 4, 5, and 7. SB431542 inhibits SMAD2/3 binding to a SMAD-responsive element at the NANOG promoter that otherwise maintains OCT4, SOX2, and NANOG expression in pluripotent cells. Using a small molecule-based method, we can generate MSC-like cells in 20 days compared to 30 days in our EB-based protocol and 40 + days with other monolayer-based protocols that require 30 days of differentiation (Boyd et al., 2009). With this method, MSCs can be generated in a rapid and complete manner without immortalization, co-culture with mouse MSCs, epitope selection, or physical selection procedures. In contrast to undifferentiated iPSCs and ESCs, iPS and ES-MSCs did not dependent on attachment factors such as gelatin, fibronectin, or Matrigel, and the feeder layer or growth factors. Following SB431542 treatment for 10 days, MSC genes were not highly expressed in ESCs or iPSCs, however, after one or two passages in MSC medium, cells acquired a MSC phenotype and function (Y. S. Chen et al., 2012). Therefore, SB431542 offers a promising strategy to differentiate human iPSCs into MSCs for use in regenerative medicine.

The isolation techniques were divided into two categories, the explant culture method and the enzymatic culture method. Explant culture is one of the best isolation methods and *in vitro* cell cultivation techniques (Mushahary et al., 2018). Although explant culture increases the initial culture time, it is an inexpensive process that requires little manipulation and produces a homogeneous cell population, as only those that can be transferred from tissue to plastic will grow (Priya et al., 2014). Enzymatic culturing technique includes an additional step that involves incubation of the cut tissues in an enzyme solution that can degrade the extracellular matrix (ECM) (Mushahary et al., 2018). Enzymatic digestion allows rapid cell separation, but enzymes increase the cost of the process and produce a very high proteolytic stress that can damage the cell membrane adhesion capacity and viability (J. Hua et al., 2013). Additionally, this technique requires multiple steps that lead to an increased risk of biological contamination. Severe tissue degradation causes cultures from enzymatic digestion to become highly heterogeneous with high hematopoietic contamination that results in low growth rates and alterations in morphology (Salehinejad et al., 2012). Besides the explant and enzymatic digestion method, a separation filter device has been utilized in the bone marrow that collects the nucleated cells within a rayon/polyethylene nonwoven fabric. These isolated nucleated bone marrow cells are directly placed into the culture vessels. In the standard *ex vivo* expansion culture condition, after two passages, about 2-3-fold more MSCs have been generated using a filter device compared to buoyant density centrifugation. Using a filter device is more effective in generating bone marrow MSCs (Mushahary et al., 2018).

Although MSCs have been isolated by explant and enzymatic techniques, some studies have been carried out to compare the main characteristics. One of the studies examined the surface expression of CD markers such as CD34, CD44, CD73, CD105, and HLA-DR in MSCs that have been obtained by the explant and enzyme method (Priya et al., 2014). It shows few differences between the MSCs that have been isolated by both methods. There is also no difference in immunogenic and immunosuppressive responses. Furthermore, MSCs did not enhance peripheral blood mononuclear cell (PBMC) proliferation and strongly inhibit lymphocyte proliferation after mixed lymphocyte reaction (MLR) (Priya et al., 2014). Compared to the enzymatic method, the explant produced more pure and less heterogeneous cells that have a high proliferation rate (Salehinejad et al., 2012). It was suggested that the higher proliferation rate of MSCs derived *via* the explant method was due to a more homogeneous cell population and less enzymatic damage. High cell viability and number can be obtained through enzymatic methods (Yoon et al., 2013). Furthermore, the use of explant culture required less time compared to the enzymatic digestion method, where collagenase takes more time to break down the tissue (W. Jing et al., 2011).

In transcriptomics, the MSC transcriptome is interrogated using large gene expression-based data (GEO datasets), which reveal significant changes in expression that result from culture expansion, hypoxia preconditioning, stimulus-directed differentiation, trans-differentiation, exposure to biologics, and coculture with other cells. A genome-wide gene expression study can provide insight into the biological nature of MSCs, their expected physiological function, and their probable role in disease pathophysiology. Understanding the data on MSC gene expression can provide insight into native physiological function, improve operational definitions of MSCs, and inform how culture conditions and clinical manufacturing protocols can best characterize their composition and function before they are administered to patients (Pittenger et al., 2019). The original purpose of MSC gene expression studies was to establish a shared identity for bone marrow-derived MSCs (BM-MSCs) *in vitro*. Serial gene expression (SAGE) was used to analyze the transcriptome of human and mouse BM-MSCs to accomplish this goal. These catalogued transcripts were found to reflect their stem/progenitor properties, as well as paracrine functions related to support and skeletal homeostasis (Tremain et al., 2001). MSCs are widely used for clinical therapies because gene expression data confirm their skeletogenic, angiogenic, anti-inflammatory, and immunomodulatory activities. Studies with RNA sequencing have provided more information on how MSCs respond to differentiation-inducing stimuli at the cellular level (Pittenger et al., 2019). A study of MSC differentiation into an adipogenic or osteogenic line found significant changes in their transcriptomes. ChIP-Seq studies showed that osteoblasts derived from MSCs resembled more closely naive cultured MSCs in terms of epigenome (Meyer et al., 2016).

### 2.2 Induced pluripotent stem cells

Before the generation of induced pluripotent stem cells (iPSCs), embryonic stem cells (ESCs) were the only source of pluripotent stem cells that had been studied. Generation of induced pluripotent stem cells (iPSCs) by reprogramming somatic cells to an ESC state with the help of a cocktail of transcription factors was a significant advancement in stem cell research (Bhartiya et al., 2013). ESCs are extracted from the inner cell mass. In 2006, Takahashi and Yamanaka reprogrammed the mouse fibroblast using the retroviral transduction method using 24 genes (Takahashi and Yamanaka, 2006). From the gene pool, four genes have been narrowed down, which are OCT4, SOX2, c-Myc, and KLF4 (Yu et al., 2007). iPSCs are usually like ESCs in morphology, expression of a gene, *in vitro* differentiation potential, and teratoma formation (Jung et al., 2012). iPS cells can differentiate into primary germ layers *invitro*, such as ectoderm, mesoderm, and endoderm (Medvedev et al., 2010).

**FIGURE 4:**
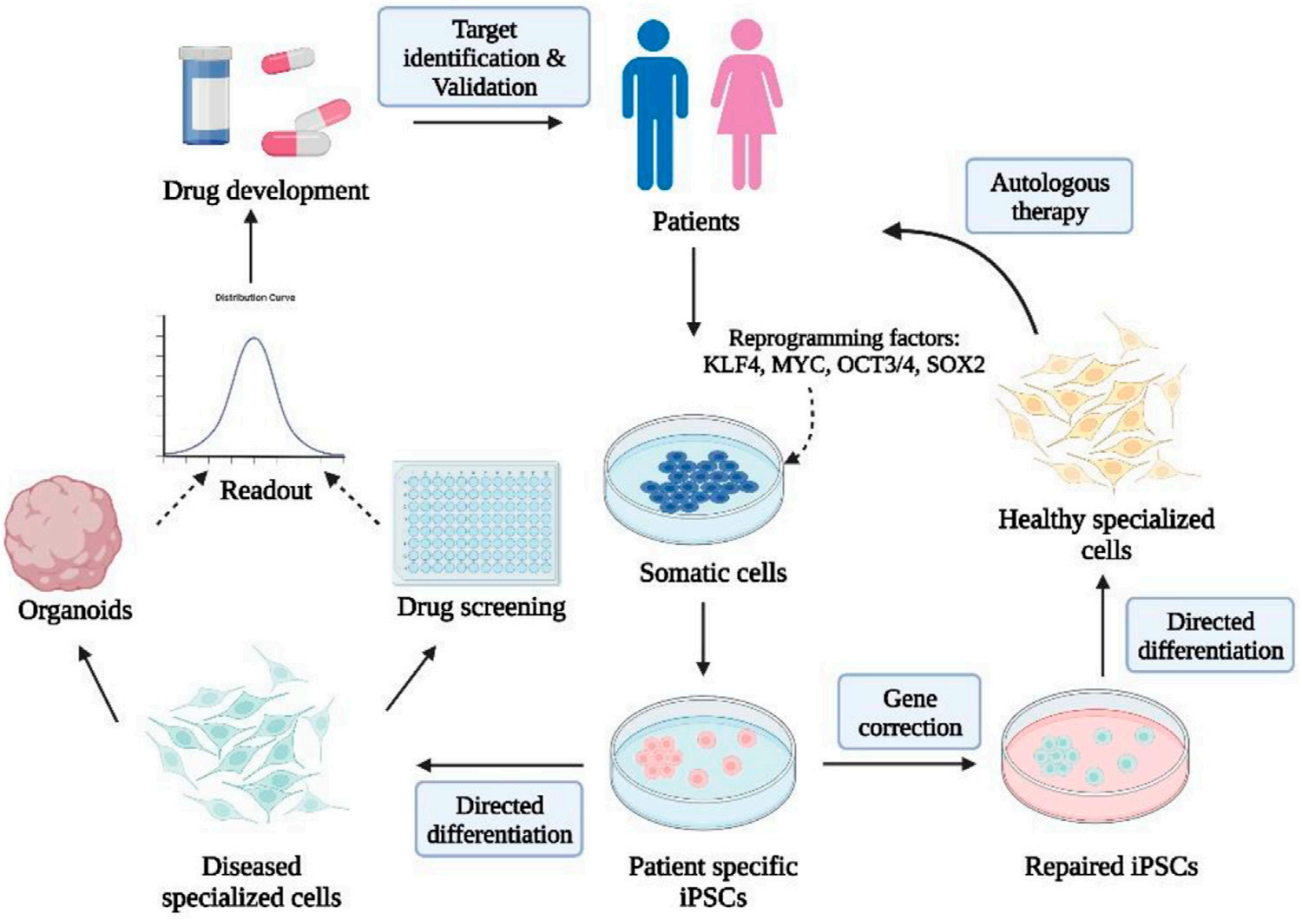
Schematic diagram of iPSC-based cell therapy. Created by Biorender.com.

One of the best studied pluripotency regulators is Oct4. Early embryonic cells, germ line cells, and cultured pluripotent stem cells express Oct4, which activates stem gene transcription. The Oct4 protein interacts not only with stemness factors such as Nanog and Sox2, but also with transcriptional repressors such as Polycomb Group proteins (Wang and Dai, 2010). Oct4 is coactivated by Sox2, which is a transcription factor (Chew et al., 2005). Binding of Oct4/Sox2 dimers to Oct4 and Nanog promoter sequences leads to upregulation of their transcription. Nanog is a homeobox-containing transcription factor that stabilizes the stem network (Masui et al., 2007). A zinc finger-containing transcription factor, KLF4, regulates the expression of Oct4, Sox2, and Nanog (Chambers et al., 2003). When Klf4 is overexpressed in ES cells, Oct4 is also induced, further increasing the self-renewal capacity (Y. Li et al., 2005). c-Myc facilitates reprogramming with greater efficiency and speed (Nakagawa et al., 2010). Post-transcriptional expression of Oct4 is stimulated by LIN28 through direct interaction with its mRNA (Qiu et al., 2010). Human protein Glis1 has been recognized as a replacement for c-Myc in recent years (Maekawa et al., 2011). A GLIS1 transactivator activates Wnt ligands, such as Lin28a, Nanog, Mycn, Mycl1, and Foxa2. Figure 4 shows the schematic diagram of iPSC-based cell therapy.

#### 2.2.1 Methods used for iPSCs reprogramming

Cell reprogramming efficiency remains low, therefore reprogramming techniques are being intensively researched to produce iPSCs, enhancing the efficiency, process, quality, and safety of cells (Belviso et al., 2020). In general, the enhancement focuses on several elements of the reprogramming method, which are the sources of somatic cells, the reprogramming factor cocktail (Al-Anazi, 2020), culturing and maintaining conditions for iPSCs and most importantly, the method for introducing the reprogramming factors.

A major concern associated with the generation of iPSCs is the use of retrovirus as a reprogramming method to achieve the incorporation of reprogramming factors into the host cell genome. This may lead to the formation of teratomas as a result of oncogene expression such as c-Myc and KLF4 (Riggs et al., 2013). Now, different types of methods are being studied to induce the expression of reprogramming factors. The methods are classified into two major components which are reprogramming *via* integrating or non-integrating systems using either viral or non-viral methods (Telpalo-Carpio et al., 2013).

#### 2.2.2 Integrating and non-integrating viral vectors

**FIGURE 5:**
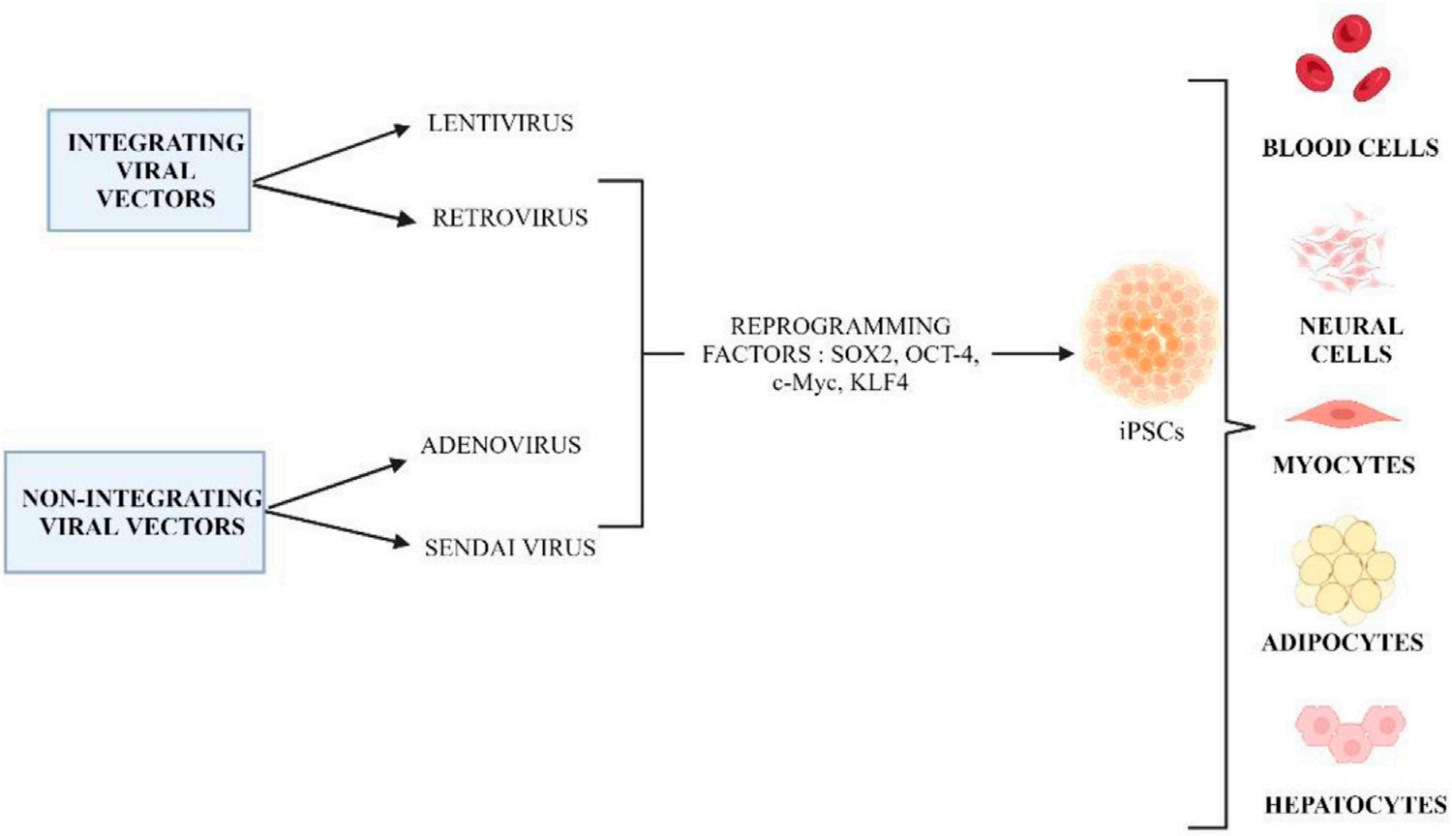
Different methods of integrating and non-integrating viral vectors are used in cell reprogramming to generate iPSCs. Created by Biorender.com.

Several types of integrated viral vectors, such as retrovirus and lentivirus, have been widely used in the generation of iPSC cells. Figure 5 shows the different methods of integrating and non-integrating viral vectors in cell reprogramming. Retroviral vectors were the first type of vector to be used to generate iPSCs, and the site of viral incorporation has been extensively researched (Han and Yoon, 2011). The Moloney murine leukemia virus (MMLV) was the first retrovirus that was used to deliver specific transcription factors to human and mouse fibroblasts. MMLV has the ability to infect actively dividing cells and remains silent in immature cells like ESCs (Han and Yoon, 2011). Retroviruses are more likely to cause malignant formation when they integrate near transcription sites.

Compared to fibroblasts, human ectodermal keratinocytes can be reprogrammed to pluripotency at least 100 times more efficiently and two-fold faster (Aasen et al., 2008). As a result, keratinocyte-derived iPS (KiPS) cells are less likely to integrate retroviruses than fibroblast-derived iPS cells, highlighting the importance of intrinsic differences between these two types of somatic cells. The presence of keratinocyte stem cells may contribute to a higher efficiency of keratinocyte reprogramming (Fuchs, 2007). Keratinocytes are more similar to hES cells and KiPS cells than fibroblasts are to hES cells and KiPS cells based on the expression of stem cell-related genes. CD24, a putative stem cell marker, was also detected in primary keratinocytes but not in fibroblasts by flow cytometry (Aasen et al., 2008).

In addition to retrovirus, the lentiviral vector (LV) is known to be more suitable due to its broad tropism. LVs are used to reprogram a wide range of somatic cells such as mice, rats, and humans (Al Abbar et al., 2020). HIV is the source of the most common lentivirus used as a delivery vector. Lentiviruses have higher cloning capacity and infection efficiency compared to retroviruses. LVs can replicate in both dividing and non-dividing cells, unlike MMLV-based. Lentivirus and retrovirus vectors carry a severe risk of insertion mutagenesis during transfection due to their genomic integration. These vectors can have a high risk of tumorigenicity when reactivated during the differentiation of iPSCs even though they have been silenced (Belviso et al., 2020). In integrating viral vectors, lentiviral is known to be better than retroviral vector. In a study researchers found lentiviral vectors did not cause higher tumor incidence or earlier tumor onset in mice compared to retroviral vectors (Montini et al., 2006). Lentivectors can overcome the limitations of retroviruses, such as their inability to transduce quiescent cells (Lewis and Emerman, 1994). As a result of their development from complex retroviruses, lentiviral vectors have several advantages over simple retroviral vectors, including higher virion stability and titer, and a reduced frequency of insertional mutagenesis (Dufait et al., 2012).

Furthermore, the next method of reprogramming involves non-integrating viral vectors such as Adenoviruses and Sendai viruses. Human and mouse iPSCs have been successfully generated using adenovirus (W. Zhou and Freed, 2009). The iPSCs showed no evidence of insertion of exogenous DNA insertion into the host genome. Human iPSCs produced from adenovirus have been shown to be pluripotent and capable of differentiation into three germ layers (Stadtfeld et al., 2008). Another study uses adenoviral vectors expressing c-Myc, KLF4, OCT4, and SOX2 to produce iPSCs from human embryonic fibroblasts, and the resulting iPSCs express ESC-specific markers and undergo significant differentiation. It showed the ability, and viral or transgenic integration was absent (W. Zhou and Freed, 2009). However, this method eliminate the risk of malignant transformation associated with retroviruses or lentiviruses and reduces the efficiency of repeated transduction to maintain an appropriate level of transgene expression. One of the factors is the time taken for stem cells to be cultured prior to transduction, where a longer period may show altered transduction of the AAV vector compared to stem cells cultured for a short period of time. Furthermore, the number of vector genomes per cell used to transduce stem cells is inconsistent. As for Sendai virus, the replication cycle is localized in the cytoplasm and it only takes a shorter time for the virus to be taken up by the cells.

An alternative to adenovirus, Sendai virus (SeV) can be used where it is an RNA virus that could infect a variety of proliferating or inactive cell and does not penetrate the host cell nucleus (Fusaki et al., 2009). They have a low risk of genomic insertion and are used to reprogram neonatal and adult fibroblasts, which makes them suitable for vehicle Yamanaka factors (Takahashi and Yamanaka, 2006). After infection, the virus replicates in the cytoplasm, and it can be washed out of the host cells through several passages. Using the Sendai virus in reprogramming can generate iPSCs without altering the genome. Sendai virus vectors are deficient in replication and their copy becomes diluted after cell division, resulting in obtaining virus-free iPSCs after 10 passages (Belviso et al., 2020). As for adenovirus, they are highly transducible, express transgenes efficiently, and have a wide range of viral tropisms. It is possible for them to infect both dividing and nondividing cells (Ura et al., 2014). There are several unique characteristics of SeV that make it suitable for immunotherapy, including the fact that its replication cycle is localized in the cytoplasm and that it takes only a short time for the virus to be taken up by the cells (Hosoya et al., 2008). SeV has a low transgene capacity compared to other viral vectors, which limits its use.

Additionally, peripheral blood contains terminally differentiated circulating T cells (hTDCTC). It was demonstrated that hiPSCs can be generated easily, efficiently, and safely within a 1-month time frame by combining activated T cell culture with temperature-sensitive mutated Sendai virus (SeV) (Seki et al., 2010). The SeV vector was used for the generation in order to prevent transgene integration (H. O. Li et al., 2000). To reduce the expression and SeV residue in the generated lines, a mutated SeV vector was used, which was temperature sensitive. At standard culture temperatures, this type of SeV vector produces weaker transgene expression and cannot proliferate. SeV has been shown to efficiently transduce and express exogenous genes in human T cells (Okano et al., 2003). Using SeV, multiple transcription factors were delivered to cells to generate iPSC from hTDCTCs, including OCT3/4, SOX2, KLF4, and c-MYC.

Generation of iPS cells using Sendai virus was carried out using two different conditions, feeder-free condition and xeno-free condition. A feeder-free condition, using Matrigel and a defined culture medium instead of a feeder layer, minimizes exposure risks from unknown exogenous factors (Kishino et al., 2015). It is possible for the xenogeneic and chemically undefined components of culture systems to pose pathogenic risks and cause immune rejection (Y. Hua et al., 2022). Matrigel adhesive proteins have become the focus of research, along with recombinant ECM proteins synthesized from human cells. During embryogenesis, laminin is a key ECM protein, and laminin-based substrates are more effective than Matrigel in adhesion, survival, and self-renewal of hiPSCs (Miyazaki et al., 2012). Gelatin nanofibers (GNFs) have recently been developed as a low-attachment substrate, similar to Matrigel for the long-term expansion of hiPSCs (L. Liu et al., 2014). In the presence of AscleStem PSC medium, ON2, GNF fully maintained hiPSCs over 30 passages without any morphological or chromosomal abnormalities. GNF-grown hiPSCs showed a higher proliferation rate (Y. Hua et al., 2022). There are several possible reasons for the spontaneous differentiation of iPS cells in culture. The main cause is the over-confluency of cultures. Therefore, colonies must be passed before reaching full confluence to avoid differentiation. The second problem is the poor quality of the ECM coating. This can be prevented by performing quality control tests on each batch of coated vessels before they are allowed to proceed with production or adding more coating solution might reduce the risk of coating failure. For all pluripotent stem cell cultures, oxygen tension should be adjusted to 3%–5% O_2_. When cell cultures are kept outside of an incubator for prolonged periods of time, they can be exposed to high fluctuations in temperature. It is also recommended to add prewarmed reagents into culture, but to prevent growth factors from losing their activity, prolonged exposure to 37°C should be limited. Differentiated cells can be removed during dissociation with EDTA-based dissociation reagents, since iPSCs will be harvested preferentially, while differentiated cells will remain attached to the current culture surface (Rivera et al., 2020).

#### 2.2.3 Integrating and non-integrating non-viral vector

**FIGURE 6:**
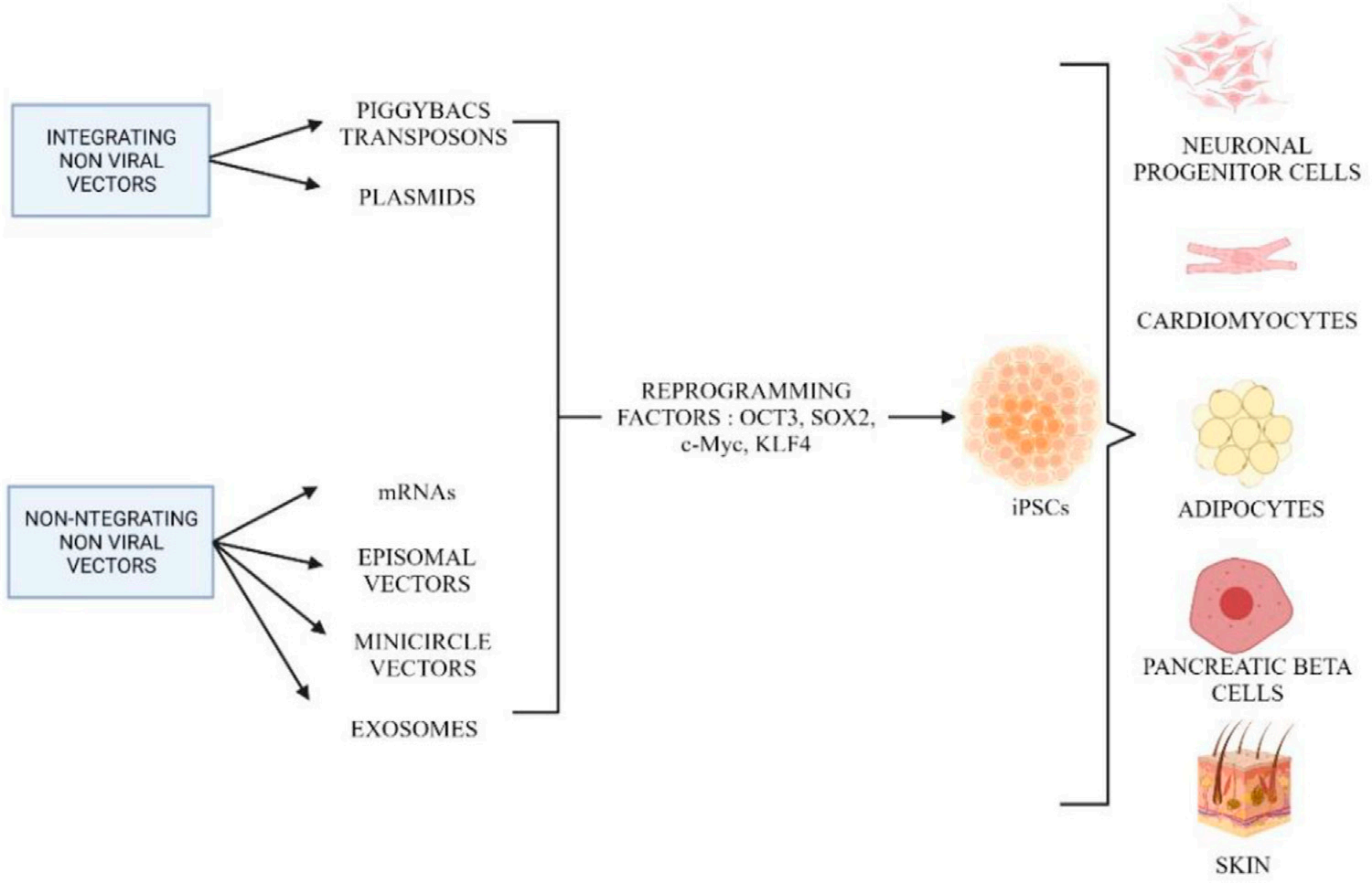
Different methods involved in reprogramming using integrating and non-integrating non-viral vectors. Created by Biorender.com.

In addition to using viral vectors, non-viral vectors are alternatives for reprogramming to produce iPSCs. Due to the limitations in integrating viral vectors, non-viral vectors are actively used that are safer for therapeutic approaches. Figure 6 shows the different methods of integrating and non-integrating non-viral vectors in cell reprogramming. Integrated non-viral vectors such as plasmids were used to generate iPSCs from mature embryonic fibroblast cells. Two plasmid constructs were used, where one plasmid encoded for c-Myc and another polycistronic vector ended the four reprogramming factors (Han and Yoon, 2011; Al Abbar et al., 2020). Furthermore, it is extremely efficient to deliver exogenous pluripotency genes using a mobile genetic element such as PiggyBac (PB) transposons. The most important and distinctive feature of this approach is that transient transposase expression can completely remove the remnants of this element from reprogrammed cells (Han and Yoon, 2011). However, the PB-like transposon elements may present in the human genome and contain endogenous, which may result in nonspecific genomic alterations following transgene excision. Using this plasmid-based protocol, plasmid-iPS cells are established at a lower rate than those induced by viruses (Okita et al., 2008). Therefore, PiggyBac transposons can be an alternative in the host genome, they can be eliminated without leaving a “footprint” of mutations. Using piggyBac transposons, it is possible to permanently eliminate reprogramming factors from iPSCs without affecting their genetic makeup (Yusa et al., 2009).

The last method used in reprogramming involves non-integrating non-viral vectors such as episomal vectors, mRNAs, minicircle vectors, recombinant proteins, microRNAs, and exosomes. These methods have been developed to generate iPSCs that are completely free of viral contamination. The use of non-replicating or replicating episomal vectors was one of the first integration-free cell reprogramming techniques. This method is very simple and does not require any manpower to perform the experiment. Furthermore, this method has a low transfection power, which requires multiple transfections to achieve a stable expression level of the desired genes. Episomes work best in reprogramming blood cells, but to generate iPSCs from fibroblasts, they need several modifications in a standard culture condition (Okita et al., 2008).

Minicircle vectors are alternatives to episomal vectors that have higher transfection efficiency. Minicircle coding genes are highly efficient in both dividing and nondividing cells and they result in a high level of desired protein expression due to being less likely to be silenced and inactivated by a cellular mechanism that targets foreign nucleic acids (Jia et al., 2010). Due to its low activation of exogenous silencing mechanisms, minicircle DNA can offer higher transfection efficiency and longer ectopic expression, making it ideal for generating iPS cells (Z.-Y. Chen et al., 2005). To examine the reprogramming capacity of the minicircle vector, we induced pluripotency in human adipose stem cells (hASCs). Compared to other types of somatic cells such as fibroblasts, hASCs can be isolated in large quantities with minimal morbidity (Fraser et al., 2006). Photon counts and qPCR confirm that minicircle DNA with the same reporter gene maintains higher expression in hASCs for a longer time than standard plasmids (Jia et al., 2010). Despite the differences in donor cell types (neonatal fibroblasts vs. hASCs) and reprogramming factors used, minicircle DNA still had higher efficiency than previous plasmid-based transfection reprogramming methods (Yu et al., 2009).

Furthermore, the use of vectors, synthetic RNA, or messenger RNA (mRNA) encoding reprogramming factors could provide a significant opportunity for the generation of integrase-free pluripotent cells. Although it requires multiple sets of transfections, this method is suitable for producing iPSCs with improved safety profiles (Warren et al., 2010). In addition, reprosomes are exosomes that contain a cocktail of reprogramming factors for a specific purpose. To generate iPSCs, exosomes appear to be suitable for clinical application, as they only need fewer procedures and carry a lower risk of tumor formation and mutation.

## 3 Advantages and disadvantages of MSCs

Mesenchymal stem cells have become the most widely used types of stem cell in therapeutic applications because of their multiple advantages. The first advantage of MSCs is being easily accessible. This can be proved by obtaining MSCs from different sources of the human body. For example, MSCs can be obtained from bone marrow, adipose tissue, human umbilical cord, amniotic fluid, dental pulp, and skin. The techniques used to isolate MSCs are minimally invasive and do not damage the patient’s organs.

Second, MSCs can be produced on larger scales, which can be used for clinical purposes. Producing therapeutic cells with good manufacturing practice (GMP) requires a scalable and controllable bioprocess that can be run in a closed system. The most widely used cell culture bioreactor on a laboratory scale, especially for anchorage-dependent cells, is the T-flask with several surface areas such as 25, 75, 150, and 225 cm^2^. T-flasks are simple to use, inexpensive, and disposable. Various types of bioreactors have been developed and utilized to scale up the expansion of hMSCs, which are multilayered cell factories, roller bottles, fixed bed bioreactors, and hollow fiber (Jung et al., 2012).

The third advantage is that MSCs have multilineage differentiation potential. MSCs can differentiate into other cell types such as chondrocytes, osteoblasts, myocytes, adipocytes, and neurons. The next advantage is that it has pleiotropic effects such as trophic properties, anti-inflammatory and immunomodulatory properties, antiapoptotic properties, and antimicrobial properties (Merimi et al., 2021). Lastly, MSCs have been widely used in clinical applications such as the treatment of neurodegenerative diseases (Boika et al., 2020), skin problems (Shin et al., 2017), diabetes wound healing (Nasiri et al., 2019), liver disorders (X. Yang et al., 2020), and cardiac ischemia (Quevedo et al., 2009; Poomani et al., 2022). Table 1 summarizes the advantages and disadvantages of MSCs.

**TABLE 1:** Summarizes the advantages and disadvantages of MSCs.

Advantages	Disadvantages
MSCs can be easily obtained from different sources of stem cells	MSCs undergo replicative senescence that arrests cell proliferation
MSCs can be produced on a larger scale for therapeutic applications	MSCs have a higher chance of malignant transformation
MSCs have a multilineage differential potential which can differentiate into other cell types	MSCs possess chromosomal aberrations and genetic instability
They required a simple procedure to isolate from the source	
MSCs are widely used in clinical applications	

In addition to these advantages, MSCs also have several drawbacks in which they experience replicative senescence, which is a senescence-related growth (Kassem et al., 1997). Replicative senescence is a common feature of the growth of diploid cells *in vitro*, limiting their ability to generate the large number of cells that require therapy. A factor that contributes to replicative senescence in MSC cells is the persistent shortening of telomeres after continuous culturing *in vitro* due to a lack of telomerase activity (Stenderup et al., 2003). This can be overcome by forced expression of the human telomerase reverse transcriptase gene (hTERT) in cultured human MSCs that can reverse the senescence phenotype and thus restore telomerase activity (Estrada et al., 2013).

Furthermore, after stem cell transplantation, the chance of getting cancer is higher. Due to their proliferative abilities, high survival rates, and resistance to apoptosis, stem cells can associate with tumor cells (Musiał-Wysocka et al., 2019). Patients who receive stem cell transplants frequently receive long-term chemotherapy, causing their immune system to malfunction, leading to an increase in the risk of cancer. Modification of MSCs and long-term *in vitro* culture can result in chromosomal aberrations and genetic instability (Barkholt et al., 2013).

In addition, tumor growth is aided by MSCs. Excessive production of cytokines such as chemokines and growth factors acts directly on the receptors on the surface of cancer cells, which controls tumor growth. The ability of MSCs to suppress the immune system contributes to the growth and spread of tumor cells (Karnoub et al., 2007).

### 3.1 Advantages and disadvantages of iPSCs

iPSCs are well established and developed, as they have several advantages in that cell sources are easily accessible for reprogramming. iPSCs can be produced by various types of somatic cells such as fibroblasts, myoblasts, keratinocytes, hepatocytes, and adult stem cells. At first, mouse embryonic or adult fibroblasts were used to produce embryonic-like pluripotent stem cells using reprogramming factors (Takahashi and Yamanaka, 2006). The use of fibroblasts as a source to produce iPSC has the advantages of being low cost, easy to use, and well established in various fields of research. Human fibroblasts can be obtained from numerous organs and tissues in the body, such as the dermal, cardiac, and lungs. Isogenic disease modelling with iPSCs can be done through two strategies. The first strategy involves repairing a pre-existing, suspected causative allele in iPSCs taken from patients who have the disease. It establishes whether this particular genetic change results in a disease phenotype, but it does not provide information on whether it is sufficient to cause the disease. In addition, patient-derived cell lines will be expected to express whatever cellular or molecular phenotypes are responsible for causing the disease, and therefore reversion of those phenotypes in the edited line can be used as an indication of the disease’s etiology. The second strategy involves taking an iPSC line from a healthy individual and then introducing a lesion that might be of significance. Using this method, we can determine whether the genetic mutation causes the disease phenotype, as it removes it from the genetic background of the diseased person (Bassett, 2017).

Next, patient and disease-specific human cells are now readily available due to recent developments in iPSCs technology (Gomes et al., 2020). Patient-specific iPSCs have been developed as a resource for drug testing, toxicity, cell replacement treatment, and disease modelling (Chun et al., 2011). For example, liver transplantation is the only therapy option for people with end-stage liver disease. Hepatocyte or hepatocyte-like cell transplantation could be a viable alternative to liver transplantation in cases of acute liver failure. Adult human hepatocytes *ex vivo* is a good choice for cellular treatment and drug testing.

In addition, human-induced pluripotent stem cells (hiPSCs) can be made from somatic cells instead of human embryos, avoiding the ethical issues associated with embryo destruction. Informed consent, health, and safety concerns of donating eggs to produce embryos by *in vitro* fertilization also influence the use of ESC (Singh et al., 2015). Since iPSCs are made from the patient’s own body somatic cells, there will be no immunorejection (Z. Jiang et al., 2014). One of those studies showed that iPSCs have been differentiated into embryoid bodies (EB). There was no evidence of increased T cell proliferation or antigen-specific secondary immunological responses after transplantation of EB derived from iPSCs (Guha et al., 2013). Furthermore, iPSCs can reduce the overall cost of clinical trials. This is because around 5,000–10,000 chemicals must be examined in preclinical trials for each drug that reaches to the market. Using iPSCs to provide toxicity information about drugs through several cytotoxicity assays could minimize the cost of clinical studies. Animal models in clinical trials do not fully match the milieu of human cells, where the use of iPSCs reduces the expenditures involved with animal models (Singh et al., 2015).

There are also some drawbacks that need more attention when generating iPSCs. Table 2 summarizes the advantages and disadvantages of generating iPSCs. First, reprogramming methods can cause tumorigenesis (Bhartiya et al., 2013). Because iPSCs are produced through retroviral or lentiviral vectors, the viral system might integrate with the host DNA. The genetic material introduced through retroviral may randomly integrate into the host genome, which can cause genetic abnormalities and the formation of teratomas (Singh et al., 2015).

**TABLE 2:** Summarizes the advantages and disadvantages of iPSCs.

Advantages	Disadvantages
Using iPSCs has fewer ethical issues	The reprogramming method can cause tumorigenesis
iPSCs reduces the chances of immunorejection in patients	iPSC reprogramming factors may associate with diseases
Cell sources are easily accessible for reprogramming iPSCs	The lack of quality assessment and variability makes iPSCs as instable
iPSCs can be used as disease models	
Reprogramming iPSCs can reduce the overall cost for clinical trials	

The next drawback is quality assessment and variability. In iPSC research, evaluation tools for evaluation are essential for the reprogramming method and the final quality of cells. There must be some experiments to ensure that all pluripotent cells have developed and are not genetically altered during the reprogramming process. When cells are derived from sick patients for autologous treatment, there is always a concern that the disease underlying mechanism may remain in the generated iPSCs and express itself in the same way. Analyzing this variation could help to determine possible somatic tissues for the production of iPSC (Singh et al., 2015). Although genome editing is extremely effective in reducing variability between patients, the process itself can introduce artefacts from both off-target mutations and clonal variability. Controlling the variability introduced by genome editing can be achieved by reintroducing disease mutations in genetically corrected patients. The introduction of mutations into a consistent WT line can also reduce the variability between individuals and during the iPSC derivation process (Bassett, 2017).

In iPSC, epigenetic memory may not necessarily influence gene expression patterns due to missing transcription factors, but is expected to affect differentiation capacity (Cahan and Daley, 2013). iPSCs might be limited in some applications, but they may also be prone to enhanced differentiation into the parental cell type, thus enabling them to generate specific cells. According to Kim et al., iPSCs derived from adult umbilical cord blood and neonatal keratinocytes have different DNA methylation profiles and differentiation potentials (K. Kim et al., 2011). iPSCs derived from each cell type showed different DNA methylation patterns, resulting in incomplete erasure of tissue-specific methylation and aberrant *denovo* methylation. Extensive passages did not eradicate these differences (K. Kim et al., 2011).

Another significant issue is the set of genes used to generate iPSCs. Because the expression of the OCT4, SOX2, KLF4, and c-Myc genes is related to the development of many malignancies. Ectopic transcription of these genes can lead to neoplastic growth of cells produced from iPSCs. Overexpression of OCT4 induces murine epithelial cell dysplasia, while expression of SOX2 causes serrated polyps and mucinous colon carcinomas (Hochedlinger et al., 2005; Park et al., 2008). Excess KLF4 expression can be found in breast tumors and c-Myc overexpression is found in 70% of human cancers (Ghaleb et al., 2005; Kuttler and Mai, 2006). Tumor development has been observed by injecting retroviral system-generated iPSCs into blastocytes, most likely due to reactivation of c-Myc.

## 4 Concerns and issues in MSCs and iPSCs

There are some concerns that should be considered when using MSCs for therapeutic purposes, such as the way MSCs are administered inside the body. In the case of tissue defects, local implantation with direct application to the wounded area is very desirable. However, because most MSCs target systemic diseases, intravenous injection of MSCs is essential. Therefore, this is related to concerns about cell biodistribution and the number of cells that reach the target wound site. Most intravenously delivered MSCs are believed to remain in the lungs, they are the first organ to be encountered before other tissues (Mastrolia et al., 2019).

Second, there is not enough clinical evidence to support the long-term safety of MSCs. Although the use of MSCs is believed to be safe, the long-term safety of the procedure is still unknown. Large-scale, controlled, and double-blind clinical trials are needed to assess cell safety before MSC transplantation becomes a common therapeutic option (Si et al., 2011).

In addition to that, MSCs have a limited expansion rate. Theoretically, MSCs can be grown *in vitro* in a standard culture flask of any size essential for the experiment. MSCs, on the other hand, hit the Hayflick limit, where a phenomenon cell can divide several times before it reaches cell death or apoptosis. After a lengthy culture period and an increase in passage numbers, the result is a significant decrease in proliferation and morphological transition from a thin spindle shape to a flattened square shape (T. Zhao et al., 2019). The properties of MSCs are also influenced by the larger-scale growth in 2D plates over a longer time. Multiple passages have caused mutation spectra to function and cells at high passages had a reduced therapeutic impact in an animal model (Q. Zhao et al., 2019).

However, the generation of iPSCs for therapeutic use also shows some concern that needs attention. The first concern is genetic material and confidential personal information (Moradi et al., 2019). iPSCs formed from any individual carry a tremendous amount of private information (DNA) that, if misused, may violate the law and privacy of individuals. Although the donor of the cell is dead, iPSCs still contain the information of its close relatives, which can be an ethical and legal concern about personal privacy. Currently, with the development of human genome sequencing data, genetic information can determine close relatives (Lin et al., 2004).

Second, it is necessary to obtain informed and voluntary consent from participants whenever an individual or their cells are used in the research (Jefford and Moore, 2008). The type of informed consent form used and the information contained inside is crucial. If patient-derived iPSCs are only used for laboratory research, they must be included in the consent form and notified to the cell donor. Researchers must take responsibility for explaining all research purposes and then discuss the possible adverse effects of treatment in patients (Moradi et al., 2019).

The next concern is the possibility of the potential tumorigenicity of undifferentiated iPSCs in the cell population. It is possible for iPSC transplanted in an undifferentiated condition to develop teratomas, as well as malignant tumors such as neuroblastoma and follicular carcinoma (Okita et al., 2008). As a result, if transplanted cells are contaminated with undifferentiated iPSC, there will be a possible risk of tumorigenicity to patient health. Different methods such as purification using Fluorescence-activated cell sorting (FACS)/magnetic bead-based sorting (MACS) have been identified to eliminate the potential risk of tumorigenicity (Sutermaster and Darling, 2019). Monoclonal antibody (mAb) raised against hESCs, designated SSEA-5, which binds to the glycan H -1 expressed at higher levels in hPSCs (Tang et al., 2011). A significant reduction in teratoma formation was observed when high cells were separated by fluorescence-activated cell sorting (FACS). In partially differentiated cultures, depletion with SSEA-5 alone greatly reduced teratoma-initiating activity (Tang et al., 2011). The only way to achieve complete removal was to combine SSEA-5 with two additional pluripotent surface markers (PSM), such as (SSEA-5, CD9, CD90 and SSEA-5, CD50, CD200).

## 5 Mechanism of action and response of MSCs and iPSCs in different environments

### 5.1 Immunomodulatory effects and stress responses in MSCs

Paracrine activity, which secretes a range of soluble molecules to exert immunomodulatory, angiogenic, antiapoptotic, and antioxidative effects, is the main mechanism behind MSC-based therapy. MSCs can adjust their immunosuppressive effect and enhance cell survival through cell-to-cell interaction (Liang et al., 2014). MSCs can be used in allogeneic transplants because of their minimal immunogenicity. MCS secrete transforming growth factor-β (TGF-β), hepatocyte growth factor (HGF), inducible indoleamine 2,3-dioxygenase (IDO), class I human leukocyte antigen (HLA) -G5, prostaglandin E2 (PGE2), interleukin (IL) -6, IL-10 and TNFα-stimulated gene protein (TSG)-6 to inhibit CD4^+^ cells CD8^+^ T cells, and dendritic cell maturation and suppress plasma cell immunoglobulin production (Fan et al., 2020). In a study, MSCs were found to decrease lymphocyte proliferation, modify lymphocyte response to allogenic target cells, and prolong skin graft survival in MHC-mismatched baboons after being delivered intravenously (Bartholomew et al., 2002). Another study showed that soluble substances produced by MSCs play an immunomodulatory role in a coculture of MSCs and T-lymphocytes in a Transwell method that eliminates cell-to-cell contact. The addition of monoclonal antibodies such as TGF-β and HGF inhibits T cell proliferation (Di Nicola et al., 2002; Liang et al., 2014).

MSCs offer novel and interesting ways to improve bone regeneration, where bone is one of the tissues in the body that can generate without leaving scars. Following bone injury, a complicated bone healing process occurs to restore bone structure and function. Locally and systemically infused MSCs traveling to the damage site at earlier stages of bone fracture repair were attracted by strong chemokines that were generated at the fracture site. The ligand of the chemokine C-X-C motif ligand 12 (CXCL12) has been found to be upregulated in this setting, stimulating MSC migration that expresses the chemokine receptor 4 (Yellowley, 2013). In an experimental femoral bone defect model, adenoviral-induced adipose tissue grafts that express CXCL12 and bone morphogenic protein 2 (BMP-2) have been used at the damage sites. The MSCs were then administered systemically after 24 h and migrated for 42 days at various intervals, and a significant increase in bone volume fraction and bone healing was found compared to the negative control. CXCL12 and BMP2 increased the recruitment of MSCs to the injury site, and osteoblasts were found to outnumber osteoclasts, indicating a proclivity to improve bone remodeling (Zwingenberger et al., 2014).

On the other hand, Alzheimer’s disease (AD) is a progressive neurodegenerative brain disease marked by memory loss and cognitive impairment. Due to the immense potential of MSCs, such as differentiation potential, immunoregulatory function, and lack of immunological rejection, MSC transplantation is becoming a popular therapy in AD. Primary immune cells in the brain, astrocytes, and microglia play a key role in neuroinflammation. The effect of hUCB-MSCs on reducing Aβ accumulation is probably due to immunomodulatory inhibition of β-site APP-cleaving enzyme 1 (BACE1) at the site. Placenta-derived MSCs (PDMSCs) reduce BACE1 expression and γ-secretase activity and improve cognitive impairment in an Aβ1-42-infused mouse model (Yun et al., 2013). hUCB-MSC transplantation in APP/PS1 double transgenic mice significantly reduced the levels of Aβ, BACE1, and tau hyperphosphorylation levels and improved spatial learning and memory impairment by targeting anti-inflammatory cytokines (IL-4, IL -10, TGF- β) and down-regulation of pro-inflammatory cytokines (IL-1, TNF- α) through activation of microglia (Lee et al., 2012). MSC therapy increased IL-4 production, which may lead microglia to produce insulin-like growth factor (IGF)-1 (Butovsky et al., 2006), reducing Aβ toxicity and enhancing Aβ phagocytosis. Systemic transplantation of hUC-MSCs into APP/PS1 transgenic mice reduced interferon-γ (IFN-γ) and increased IL-10 and transforming growth factor (TGF-1) in peripheral plasma, significantly improved cognition defects and reduced Aβ plaque deposition (H. Yang et al., 2013).

Astrocytes have many chemokines and inflammatory cytokine receptors, including IL-1β and TNF-α (Morales et al., 2014). Astrocytes are critical for Aβ removal and breakdown when stimulated by IL-1β and TNF-α. MSC transplantation was found to help neonatal rats with hypoxic ischemic brain injury recover their learning and memory function by reducing reactive astrocyte growth (He et al., 2019). Based on the findings, exogenous MSCs play a restorative role in AD by decreasing astrocyte activation. When MSCs are placed in the microenvironment of wounded tissues, they can release different substances such as TGF- β and IGF-1 that cause activation (L. Zhang et al., 2020). Subsequently, activated astrocytes can remove Aβ plaque deposition and secrete TGF-β and IGF-1 as neuroprotective agents. MSCs are linked to Toll-like receptors (TLRs), which allow MSCs to secrete IL-10 and TGF- β1 (DelaRosa and Lombardo, 2010). When inflammatory cytokines released by MSCs engage TLRs, such as TLR3, they induce a comprehensive neuroprotective response by increasing the production of anti-inflammatory cytokines such as IL-9, IL-10, and IL-11 while downregulating proinflammatory cytokines such as IL-12 (Bsibsi et al., 2006).

Mesenchymal stem cells have been exposed to stresses mainly due to oxidative stress. In the context of oxidative stress, reactive oxygen, and nitrogen species (ROS and RNS) are produced unregulated and/or scavenged. In MSC differentiation, ROS are predominantly generated by mitochondrial complexes I and III and NADPH oxidase isoform NOX4 (Schröder et al., 2009). All biomolecules can be damaged by free radical accumulation, including DNA, proteins, and lipids. To maintain cellular proliferation, differentiation, and survival, ROS levels must be kept at a low level to prevent cell damage and dysfunction (Atashi et al., 2015). Initially, MSCs exhibit little ROS and high glutathione levels, but other studies indicate that they lack antioxidant activity and are much more susceptible to oxidative stress than differentiated cells (Ko et al., 2012). A high level of ROS or exogenous H2O2 can inhibit the self-renewal, differentiation, and proliferation of MSCs. However, antioxidants stimulate MSC proliferation (Zou et al., 2004).

In most studies, ROS have been found to inhibit osteogenic differentiation. The addition of exogenous H2O2 reduces osteogenic differentiation in human and murine MSCs, as well as osteoblast precursors (C.-T. Chen et al., 2008). Furthermore, the osteogenic potential is decreased in older donors’ MSCs (de Girolamo et al., 2009). Human MSCs that are induced to osteogenesis *in vitro* have been shown to upregulate the number of copies of mtDNA, respiratory enzyme proteins, superoxide dismutase 2 (SOD2, alias MnSOD), the consumption of catalase oxygen and antioxidant enzymes, but a decrease in ROS. Furthermore, undifferentiated MSCs produce greater amounts of glycolytic enzymes and lactate. Compared to MSC-differentiated osteoblasts, which rely more on mitochondrial oxidative metabolism for energy, MSCs use more glycolysis for energy.

An increase in ROS occurs during adipogenesis as MSCs differentiate into adipocytes. Human MSCs upregulate antioxidant enzymes such as SOD, catalase, and GPX during adipogenesis (Higuchi et al., 2013). The addition of exogenous H2O2 or ROS induces adipogenesis in human and murine MSCs and adipocyte precursor cells, supporting the idea that ROS contribute to adipogenesis (Schröder et al., 2009). Furthermore, H2O2 increases adipogenesis at higher doses, as adipogenesis is dose dependent. Consequently, N-acetylcysteine (NAC), a ROS scavenger, inhibited adipogenesis in the mouse MSC cell line 10T1/2 (Kanda et al., 2011). Furthermore, ROS generated by mitochondrial complex III are essential for activating adipogenic transcription factors. Adipogenesis increases mitochondrial biogenesis and oxygen consumption similarly to osteogenic differentiation (Wilson-Fritch et al., 2003).

Primary murine chondrocytes and the ATDC5 cell line require ROS generated by NADPH oxidases two and four for chondrogenic differentiation (K. S. Kim et al., 2010). Chondrogenesis was associated with a reduction in SOD3 levels (Nightingale et al., 2012). SOD3 is known to help inhibit ROS production in the extracellular matrix. Furthermore, ROS removal with NAC blocked the differentiation of chondrogenic cells. The increase in ROS levels stimulated chondrocyte hypertrophy, which was inhibited by NAC (Morita et al., 2007).

### 5.2 Stress responses in pluripotent stem cells

As with MSCs, iPSCs have effects on immunomodulation where they have greater immune privilege, they have a higher survival rate, and the graft was better following transplantation. This suggests that iPSCs have a greater advantage in allogeneic transplantation without immunological rejection. As growth factors, hormones, culture environment, and 3D structure affect pluripotent stem cells, various stress-inducible mechanisms have been discovered to increase pluripotent stem cells into specific lineages. To preserve the normal function of cells, they respond to a range of stimuli posed by the environment, known as cellular homeostasis that includes hypoxic, oxidative, thermal, mechanical, physical, and metabolic.

Hypoxia is caused by a reduction in cellular oxygen. Hypoxic stress is defined as the occurrence of a molecular event in response to hypoxia in less than 5% (Sridharan et al., 2017). When a cell is exposed to hypoxia, transcription factors known as hypoxia-inducible factors (HIFs), such as HIF-1α, HIF-2α, and HIF-1β, launch a cascade of hypoxic signaling (Sridharan et al., 2017). HIFs play a key role in pluripotency and proliferation of pluripotent stem cells. Maintaining human ESCs at low oxygen tension of 2%–5% helps reduce spontaneous differentiation, improve proliferation, and promote the expression of critical pluripotent markers (H-F. Chen et al., 2010). HIF-2α is seemed to be more prevalent than HIF-1α in signaling. HIF-2α has been shown to influence the expression of OCT4 and NANOG (Forristal et al., 2010; Petruzzelli et al., 2014).

Next, there is the thermal stress for directed differentiation. An elevated temperature greater than 40°C promotes protein denaturation and aggregation, resulting in cell toxicity and cell death (Y. E. Kim et al., 2013). Heat shock factor (HSF1) is activated in response to cell stress and increases the transcription of the gene that encodes molecular chaperons. The heat shock response (HSR) is normal in pluripotent stem cells, and it has been proven that human and mouse ESCs are resistant to high temperatures compared to differentiated cells (Prinsloo et al., 2009). Furthermore, temperature stress at 42°C was discovered to alter gene expression by activating the decommissioning of their enhancers, which is mediated by pluripotent factors such as KLF4, NANOG, OCT 4, HSF1 and AP-1 (Lyu et al., 2018). Heat shock treatment at 46°C promoted the differentiation of human ESCs through HSF1-mediated suppression of OCT4 expression. HSF1 inhibits OCT4 expression, and SAPK/JNK mediates this impact through phosphorylation (Byun et al., 2013).

In addition to that, mechanical forces have been discovered to modulate a variety of physiological processes. Cell growth, differentiation, shape changes, and cell death are all known to be regulated by reaction to mechanical forces (Weng et al., 2016). Fluid shear stress and a signal of stiffness are the two mechanical forces involved in stem cell activity and differentiation (Kaitsuka and Hakim, 2021). Pluripotent stem cells use the mechanosensitive ion channel Piezo1 and its primary cilium that regulates mechanotransduction. It was discovered that a stiff substrate stimulates focal adhesion *via* the activation of focal adhesion kinase (FAK) and steroid receptor coactivator (SRC). Then FAK phosphorylates and activates YAP, resulting in activation of the YAP/TAZ transcription factor and nuclear translocation, which is known to be involved in cellular mechanoresponses (Dupont et al., 2011). It was discovered that YAP binds to the promoters of pluripotent genes in pluripotent stem cells and is essential for the pluripotency of mouse ESCs.

## 6 MSC disease model

MSCs were first used in tissue injury research due to their multipotent capability. MSCs have been studied and have found to have several properties that can help them for therapeutic purposes. Examples of diseases that have used MSCs as therapeutic potential include bronchiolitis obliterans, Alzheimer’s disease, retinal ischemia, Type 2 diabetes mellitus, schizophrenia, *etc.* Table 3 summarizes the list of diseases that have been studied using MSCs.

**TABLE 3:** Shows the diseases that have been studied using MSCs.

MSC sources	Diseases	Key findings	Key molecules	Signalling pathway involve	Animal model	References
Bone marrow mesenchymal stem cells (BM-MSCs)	Bronchiolitis obliterans	Three different administrations involve endotracheal (ET), intravenous (IV), or lung injection (LI). As BMSCs differentiate into alveolar cells, they modify bronchiolitis obliterans in a positive way. BMSC administration of BMSCs by endotracheal infusion 7 days after heterotopical tracheal transplant could be an effective way to prevent BO in this animal model	Surfactant protein B, which is a specific biomarker for alveolar epithelial cells	N/A	Wistar rats	Gómez de Antonio et al. (2020)
Adipose-derived mesenchymal stem cells (AD-MSCs)	Cognitive problems in Alzheimer’s disease	After intravenous transplantation, AD-MSCs and MT-AD-MSCs migrated to brain tissue. Pretreatment of AD-MSC with melatonin helps improve learning, memory, and cognition compared with AD-MSC.	Well-known anti-inflammatory and antioxidant agents Melatonin were used to pretreat AD-MSCs	MT exhibits a major ROS elimination potential, inhibits the p53 pathway, and regulates intracellular signaling	Wistar rats	Nasiri et al. (2019)
Mesenchymal stem cell-derived extracellular vesicles (MSC-EVs)	Retinal ischemia	In a rat model of retinal ischemia, administration 24 h after the event, increased functional recovery and neuroinflammation and apoptosis were decreased. In the retina, EVs were taken up by microglial cells, retinal ganglion cells, and neurons. A saturable binding to vitreous humor components was observed for 4 weeks after intravitreal administration. Reduce cell death and attenuated loss of cell proliferation	Caveolar endocytic pathway mediated by cell surface HSPG receptors	Caveolar pathways, phagocytosis, and macro-pinocytosis involved in endocytosis of EVs	Wistar rats	Mathew et al. (2019)
Exosomes derived from human umbilical cord mesenchymal stem cell-derived exosomes (hUCMSC-ex)	Type 2 diabetes mellitus	Intravenous injection of hucMSC-ex reduced blood glucose levels. In T2DM, HucMSC-ex restored insulin receptor substrate 1 and protein kinase B phosphorylation (tyrosine site), increased muscle glucose transporter 4 expression, and increased liver glycogen storage to maintain glucose homeostasis by increasing glycogen storage. HucMSC-ex inhibits STZ-induced apoptosis, relieving T2DM of insulin-secreting functions	Insulin resistance is characterized by activation of protein kinase B (AKT) in patients with T2DM.	Insulin signaling pathway activation and IRS-1 tyrosine phosphorylation play key roles in glucose transport and metabolism	Sprague-Dawley (S-D) rats	Sun et al. (2018)
Exosomes derived from bone marrow mesenchymal stem cell derived exosomes (BMMSC-ex)	Bone regeneration	Cytokines and growth factors promote bone regeneration during the early stages, as well as enhanced angiogenesis. Histological analysis showed that in the MSC-Exo group, the newly formed bone area was larger than in the MSC-CM group	Growth factors such as insulin growth factor-1 (IGF-1), vascular endothelial growth factor (VEGF), and transforming growth factor-1 (TGF-1) stimulate bone regeneration	MiRNAs enhance angiogenesis and osteogenesis	Wistar rats	Takeuchi et al. (2019)
Human endometrial mesenchymal stem cells (eMSCs)	Asherman’s syndrome (AS)	Rats modeled AS had significantly improved fertility when eMSCs were administered intrauterine in spheroids	Tumor necrosis factor-induced protein 6 (TSG-6) is an anti-inflammatory protein, while the hepatocyte growth factor (HGF) protein promotes angiogenesis and anti-apoptosis, and prostaglandin E receptor (EP2) interferes with immune stimulation	N/A	Wistar rats	Domnina et al. (2018)
Mesenchymal stem cell-derived extracellular vesicles (MSC-EVs)	Schizophrenia	MSC-EVs improve schizophrenia-like behaviour. MSC-EVs administered intranasally improve social interaction and disrupt prepulse inhibition (PPI) in mice treated with PCP. PCP-treated mice were found to have lower glutamate levels in their CSF when treated with MSC-EV, which may explain the reduced toxicity	Parvalbumin shows a decrease in schizophrenia mice at the level of prefrontal cortex (PFC) schizophrenia mice	N/A	C57Bl/6J mice	Tsivion-Visbord et al. (2020)
Human umbilical cord mesenchymal stem cells (hUC-MSCs)	Atherosclerosis	Reduces the burden of atherosclerotic plaques by reducing inflammation, regulating the intestinal flora, and repairing the damaged endothelium. With UCSC transplantation, the pathological inflammatory response was diminished by inhibiting cell apoptosis in susceptible plaques, reducing macrophages, and increasing anti-inflammatory cytokines. Transplantation of UCSCs improves peripheral blood vessel morphology and function during the early stage of AS.	Trimethylamine N-oxide (TMAO) is a microbial-dependent metabolite that increases the atherosclerosis lesion by increasing the expression of scavenger receptors	N/A	Rabbit	(Y. Li et al., 2021)
Embryonic stem cell-derived mesenchymal stem cell (ES-MSCs)	Osteoarthritis	Decelerates the progression of OA in a rat model, and multiple injections were required for long-term improvement	N/A	N/A	Sprague Dawley rats	Xing et al. (2021)
Human embryonic stem cell-derived mesenchymal stem cells (ES-MSCs)	Canine anal furunculosis	This is the first study to demonstrate the safety and potential therapeutic efficacy of hESC-MSCs in a large animal model. Intralesional injection of hESC-MSCs after 3 months and 6 months completes the fistula closing. Using this canine model, researchers can investigate the mechanisms of stem cell-derived therapies and evaluate how they are administered, dosage, frequency, and safety	IL-2 plays a key role in T cell activation/proliferation, so lowering circulating levels of this cytokine may reduce autoreactive T cell proliferation. IL-6 is one of the most common pleiotropic cytokines associated with inflammation and fistulas on human skin	N/A	Dogs	Ferrer et al. (2016)

Human umbilical cord-derived mesenchymal stem cell exosomes (hucMSC-ex) have been studied for their therapeutic effect on type 2 diabetes mellitus (T2DM). T2DM is mainly caused by peripheral insulin resistance, loss of pancreatic β-cell mass, and cell malfunction. This may lead to an uncontrolled glucose level in the body. T2DM is currently treated with daily insulin injection and with drugs such as metformin and thiazolidinediones (Nyenwe et al., 2011). In this study, exosomes have some active contents that can mediate their therapeutic effect on diabetes. They hypothesized that, like hucMSC, hucMSC-ex could reduce hyperglycemia in T2DM. This study tested the feasibility and effectiveness of using hucMSC-ex to reduce T2DM in a rat model. This rat model has been induced by a high-fat diet (HFD) and streptozotocin (Reed et al., 2000). The result suggests that intravenous injection of hucMSC-ex can lower the blood glucose level in rats with T2DM (Sun et al., 2018). This is due to the improved insulin sensitivity in peripheral organs and decreased islet destruction. In summary, hucMSC-ex has been found to successfully treat hyperglycemia in HFD/STZ-induced T2DM rats by improving insulin sensitivity, increasing glucose uptake, and metabolism in peripheral tissues.

Furthermore, in T2DM models induced by high-fat diet (HFD) and streptozotocin (STZ) administration, human umbilical cord MSCs (UCMSC) and Wharton jelly MSCs (WJ-MSC) have been shown to improve islet cell function (J. Chen et al., 2021). It also helps restore the islet structure and prevents pancreatic cell hypertrophy and cell death. As a result, the blood level was normalized and enhanced glucose levels and insulin levels. Systemic transplantation of human adipose-derived MSCs (ADMSCs) decreased the expression of proinflammatory markers.

In another recent study, small extracellular vesicles derived from mesenchymal stem cells (MSC-sEV) improve pancreatic cell function, while, after transplantation, MSC-sEVs can chemoattract and migrate to the damaged site (F.-X.-Z. Li et al., 2021). This is due to the proliferation of cells in damaged islets. Sabry et al. discovered that injecting MSC-sEVs into STZ-induced diabetic rats caused the blood glucose level to decrease, plasma insulin levels increased, and the number and size increased (Sabry et al., 2020). MSC-sEVs improve the survival rate and function of islet cells as well as parental MSCs.

Furthermore, MSCs have the potential to treat bronchiolitis obliterans (BO), which is a fibroproliferative disease that causes inflammation in the submucosal cavity and fibrosis of the bronchiolar wall that blocks the lumen (King, 1989). Due to immunomodulatory properties, MSCs locally and systematically MSCs can decrease the progression of BO in different animal models. This study was carried out to investigate the effect of allogeneic adult bone marrow-derived mesenchymal stem cells (BM-MSC) with different ways of administration in a rat model of BO.

The route of administration of BM-MSCs to rat models includes endotracheal, intravenous, and lung injection. On day 7, rats that received BM-MSC through the endotracheal route exhibited no signs of inflammation. MSCs exhibit immunomodulatory capabilities that prevent inflammatory responses. Histopathological changes were observed in the early stages corresponding to inflammatory activity (Gómez de Antonio et al., 2020). The reparative effects of MSCs are activated by endothelial cells and fibroblasts to repair the injured site by releasing immunoregulation and growth factors.

BM-MSCs mediate the response to the injured, which develop alveolar tissues that resemble normal tissue. In summary, BM-MSCs can recognize the injury site and alter the histopathology of BO lesions and restore structure. Although intravenous and locally injected BM-MSCs have an impact on the treatment of BO lesions, endotracheal injection has the most expected results (Gómez de Antonio et al., 2020).

In another study, MSCs were used as an alternative after treating patients with BOS with allogeneic hematopoietic stem cell transplantation (allo-HSCT). Compared to non-MSC treatment, MSC infusion was associated with a considerably improved response, including an improvement in forced expiratory volume in 1 s (FEV1) and steroid reduction at 3 months. Multiple infusions of MSCs were well tolerated, with no increased risk of infection or relapse of leukemia. The efficacy of steroids and azithromycin combined with MSC was much higher than that of steroids and azithromycin alone, with 71% of MSC patients responding compared to 44% of non-MSC patients (S. Chen et al., 2019).

Furthermore, placenta-derived human mesenchymal stem cells (PMSC) and PMSC-conditioned medium (PMSCCM) slow the progression of BO by protecting epithelial integrity at the cellular level. A single injection of PMSCCM could lower proinflammatory cytokines, resulting in decreased infiltration of inflammatory and immune cell infiltration. On the third day, PMSCs were injected intratracheally, which is an important moment for epithelial regeneration. On the 14th day after tracheal transplantation, intratracheal injection of PMSCs and PMSCCM dramatically reduced CD3 + T cell infiltration. These findings imply that PMSCs and PMSCCM can have immunosuppressive properties during the cellular infiltration phase of BO formation (Y. Zhao et al., 2014). Table 4 highlights current clinical trials of the use of MSCs in cell therapy applications.

**TABLE 4:** Highlights current clinical trials of the use of MSCs in cell therapy applications.

Clinical trial identifier	MSCs source	Title	Objective	Phase	Status	Trial duration	Country
NCT04318600	Human Amniotic Mesenchymal stem cell (HA-MSC)	Allogeneic Amniotic Mesenchymal Stem Cell Therapy for Lupus Nephritis	To evaluate the safety and efficacy of human amniotic mesenchymal stem cells (hA-MSC) for the treatment of lupus nephritis (LN)	Phase 1	Completed	01 January 2014–01 January2019	N/A
NCT03333681	Mesenchymal stem cells	Evaluation of the effects of stem cell therapy on the immune response in Rheumatoid Arthritis Patients	Evaluation of the effects of mesenchymal stem cell therapy on cellular and humoral immune responses in patients with refractory rheumatoid arthritis (RA) patients	Phase 1	Completed	20 June 2016–15 September 2018	N/A
NCT03343782	Bone Marrow-derived Mesenchymal Stem Cells	Outcomes of expanded autologous bone marrow-derived mesenchymal stem cell therapy in Type II Diabetes (ASD2)	To evaluate the safety and effectiveness of autologous bone marrow-derived mesenchymal stem cell transplantation in the treatment of patients with type 2 diabetes mellitus	Phase 1and 2	Completed	01 November 2017–01 August 2019	Vinmec International Hospital, Hanoi, Vietnam
NCT02181712	Bone Marrow-derived Mesenchymal stem cells	Mesenchymal stem cell therapy for Lung Rejection	To assess the safety and feasibility of mesenchymal stem cell therapy in patients with transplant-related bronchiolitis obliterans syndrome (BOS)	Phase 1	Completed	July 2014–12 August 2021	Florida, United States
NCT03326505	Umbilical Cord derived Mesenchymal Stem Cells (MSCs)	Allogenic Mesenchymal Stem Cells and Physical Therapy for MS Treatment	To study the effect of UC-MSCs in Multiple Sclerosis and comprehensive supervised physical therapy	N/A	Completed	25 September 2017–25 January 2020	Jordan
NCT04713878	Mesenchymal stem cells	Mesenchymal stem cell therapy in patients with COVID-19 Pneumonia	Assess the effect of MSCs in providing an immune response and repairing the damaged organs	N/A	Completed	08 May 2020–30 June 2020	University of Health Sciences Istanbul, Turkey
NCT01809769	Adipose Tissue Derived Mesenchymal Stem Cells	Autologous adipose tissue-derived mesenchymal stem cell therapy for patients with Knee Osteoarthritis	To evaluate the effects of AD-MSCs in patients with knee osteoarthritis	Phase 1and2	Completed	March 2013- March 2015	Shanghai, China
NCT02611167	Mesenchymal stem cells	Allogenic bone marrow-derived mesenchymal stem cell therapy for Idiopathic Parkinson’s Disease	To evaluate the safety, feasibility, and efficacy of intravenous allogeneic bone marrow-derived mesenchymal stem cell therapy (MSC) for idiopathic Parkinson’s disease (iPD)	Phase 1	Completed	01 November 2017–18 September 2019	United States
NCT02192749	Allogeneic Umbilical Cord Mesenchymal stem cell	Allogeneic umbilical cord mesenchymal stem cell therapy for Autism	Assess the safety, feasibility, and efficacy of human umbilical cord tissue-derived stem cells that will induce a therapeutic effect in autism patients	Phase 1and2	Completed	July 2014- August 2017	Stem Cell Institute
							Panama City, Panama

### 6.1 Components expressed by MSCs in cell therapy applications

MSCs isolated from sources such as bone marrow, adipose tissue (ASC), and Wharton’s jelly possess different surface expression markers, chemokines, proinflammatory proteins, and growth factors that aid in cell therapy applications. Considering their high potential in regenerative medicine, WJ-MSCs may exhibit relatively high expression of CD54 and CD146. In response to proinflammatory mediators, CD54 (ICAM-1) stimulates the mobilization and transendothelial migration of circulating cells into injured tissues (L. Yang et al., 2005). In MSCs, CD146 (MCAM, MUC18) is a cell adhesion molecule with a higher differentiation potential (Russell et al., 2010). Both BM-MSCs and AD-MSCs express a high cell marker such as CD73, CD90, CD105, while less expression of hematopoietic markers such as CD34, CD45, and HLA-DR (Mohamed-Ahmed et al., 2018). Both BMSCs and ASCs express Stro-1 (De Ugarte et al., 2003; Strem et al., 2005), a marker of multilineage potential (Dennis et al., 2002). The study confirmed that BMSCs expressed Stro-1 more than ASCs (Mohamed-Ahmed et al., 2018). In this study, ASCs were shown to continue to increase over the next 21 days in this study. In MSCs, CD34 is believed to play an important role in their long-term proliferation. Therefore, the continued proliferation of ASCs may be related to the expression of CD34 by ASCs (Sidney et al., 2014).

As part of the IL-1 family of cytokines, IL1-RA is a receptor antagonist that belongs to at least 11 inflammatory mediators (Amable et al., 2014). Since IL1-RA has protective effects, it has already been proposed as a therapeutic candidate for the treatment of diabetes mellitus II treatment. A model of induced diabetes mellitus showed that IL1-RA prevented pancreatic mononuclear cell infiltration, islet destruction, and hyperglycemia (Sandberg et al., 1994). Ortiz et al., 2007, demonstrated in preclinical studies in mice that BM-MSCs were more effective than recombinant IL1-RA in reducing inflammation and inhibiting fibrosis (Ortiz et al., 2007). However, BM-MSC secreted detectable amounts of VEGF-D as part of the angiogenic process. As the strongest angiogenic and lymphangiogenic VEGF isoform, VEGF-D has been tested in phase I clinical trials for myocardial infarction (Rissanen et al., 2003). Genetically engineered MSCs that overexpress VEGF have been shown to overexpress VEGF are more effective in treating acute myocardial infarction than MSCs alone because VEGF extends the lifespan of MSCs, protects them from apoptosis, and improves their ability to recover heart function (Augustin et al., 2013). The highest concentrations of thrombospondin-2 were found in WJ-MSC supernatants, the most abundant angiogenic factor (Amable et al., 2014). According to Jeong and others, WJ-MSC was able to regenerate cartilage and this effect was mediated by thrombospondin-2 because thrombospondin-2 alone can exert similar effects, and knockdown of thrombospondin-2 with siRNA abolished the ability to regenerate cartilage (Jeong et al., 2013). AT-MSCs also secrete a high concentration of thrombospondin-2, making them a good candidate for cartilage regeneration, especially if patients did not cryopreserve their own umbilical cord cells and could use autologous adipose tissue (Amable et al., 2014). Compared to MSCs alone, genetically modified MSCs expressing angiopoietin-1 were more efficient in regenerating myocardial tissue after infarction (Paul et al., 2012).

MiRNAs are one of the components that were secreted out of the exosomes by stem cells. Studies have indicated that miRNAs can act on gene enhancers within the nucleus to activate genes and promote their expression (Xiao et al., 2016). This shows that miRNAs in MSC exosomes could be used as a future therapeutic target to treat diseases. There are different types of miRNAs that target different genes and can have an impact on different diseases. Using miRNAs, inflammation can be reduced and fibrosis can be prevented (W. Zhang et al., 2019). MiRNAs can be used by stem cell-derived exosomes to correct immune disorders in organ tissues, including allergic airways, Duchenne muscular dystrophy, and myocardial ischemia-reperfusion injury (Bier et al., 2018; Fang et al., 2020; J. Zhao et al., 2019). MiR-146a-5p regulates allergic airway diseases by decreasing the expression of interleukin-9 (IL-9) and interleukin-13 (IL-13) in innate lymphoid cells of Group 2. Therefore, miR-146a-5p expression in MSC exosomes is associated with allergic diseases. An ischemia-reperfusion injury causes inflammation in the heart due to the process of ischemia and reperfusion. According to these findings, miRNAs in exosomes can regulate the immune response by changing macrophages from M1 to M2 anti-inflammatory phenotypes (J. Zhao et al., 2019). In sepsis, miR-233 in MSC exosomes may protect the heart from inflammatory cytokines and reduce heart stimulation and damage (Wang et al., 2015).

### 6.2 Disease model of iPSCs

MSCs are the most established stem cells that have been used to study disease models compared to iPSCs for many decades. Human iPSCs are currently emerging in research fields and being studied in disease models. Examples of diseases that have used iPSC are Parkinson’s disease, myocardial infarction, mitochondrial DNA depletion syndrome, and Post-traumatic stress disorder (PTSD). Table 5 summarizes the list of diseases that have been studied using iPSCs.

**TABLE 5:** Shows the diseases that have been studied using iPSCs.

IPSC	Diseases	Key findings	Key molecules	Signalling pathway involve	Animal model	References
iPSC-derived dopaminergic progenitor cells	Parkinson’s disease	No tumorigenicity and toxicity of cells. Rats lesioned with 6-hydroxydopamine (6-OHDA) exhibited improved behaviour when treated with DAPs	As dopaminergic progenitors (DAPs) cells mature and become DA neurons, they generate dopamine, which improves the abnormal behavior of 6-OHDA-lesioned rats	N/A	NOG mice	Doi et al. (2020)
Human Induced-pluripotent Stem-cell–derived Cardiac Cells	Myocardial infarction	hCMP transplantation has been shown to be associated with significant improvements in lentiviral vector function, infarct size, myocardial wall stress, myocardial hypertrophy, and reduced apoptosis in the peri-scar boarder zone. A hCMP transplant also reversed some phosphorylation changes of sarcomeric regulation proteins resulting from MI. hCMP exosomes showed cytoprotective properties that promoted cardiomyocyte survival	Exosomes released from hCMP reduce myocyte apoptosis *in vitro*. They may carry paracrine factors that contribute to proliferation and angiogenesis	N/A	Porcine or pig	Gao et al. (2018)
Human Induced-pluripotent Stem-cell-Derived Hepatocytes	MtDNA depletion syndrome	DGUOK-deficient hepatocyte cells responded to NAD by activating the gamma coactivator 1 alpha (PGC1 α).ATP production was also enhanced by NAD treatment in MTDPS3-null rats and in hepatocyte-like cells lacking ribonucleoside-diphosphate reductase subunit M2B (RRM2B)	NAD acts as a lead drug that maintains mitochondrial function and restores ATP without decreasing the MtDNA copy number	NAD homeostasis plays a vital role in cellular energy metabolism	DGUOK deficient rats	(R. Jing et al., 2018)
			NAD precursors, such as nicotinamide and nicotinamide mononucleotide (NMN), help to increase the NAD^+^ level			
Human Induced-pluripotent stem-cell-derived neural progenitor cells (iPSC-NSCs)	Post-traumatic stress disorder (PTSD)	Compared with the control group, iPSC-NPCs differentiated into neurons that replaced the loss of hippocampus neurons, and their transplantation increased the expression of glial fibrillary acid protein (GFAP) and NeuN levels. NPC transplantation produced neurotrophic effects and improved behavior outcomes in cerebrovascular diseases. iPSC-NPC transplantation could induce neurogenesis and increased expression of BDNF in the hippocampus	The glial fibrillary acid protein (GFAP) maintains the mechanical strength of the astrocytic processes, and it thickens them and elongates them	N/A	Male Sprague-Dawley rats	(Q. Liu et al., 2021)
			BDNF acts as both a neurotrophic and a neuroprotector, which makes it an important modulator of the acute response to neurotrauma			

First and foremost, Parkinson’s disease (PD) is the second most prevalent progressive neurodegenerative disorder in the world that affects 0.3% of the global population over the age of 70 years (Lee and Gilbert, 2016). The development of Lewy bodies (LBs) or Lewy neurites that are positive for α-synuclein (α-syn) is the key pathological hallmark of PD. DA neurons derived from the midbrain iPSC are greatly desired for the *in vitro* PD model (Spathopoulou et al., 2022). This model could aid in studying the α-syn-driven which occurs mostly in dopaminergic (DA) neurons in the SNpc in Parkinson’s disease. This reveals that α-synuclein pathology has adequate seed quality in neurons of patients with PD induced by genetic abnormalities (Hu et al., 2020).

In the latest study, a primate received neurotoxic MPTP, which causes a PD-like condition in the host. This is due to the study on IPSC graft on the effect of the DA neuron IPSC graft (Jarbæk Nielsen et al., 2020). The primate showed a significant increase in spontaneous movement after transplantation, measured by score-based and video-based studies. For more than 2 years, no malignancies grew and only a small immune response was evoked. The capacity of patients to use iPSC from their bodies reduces the risk of immune-mediated graft rejection and eliminates the requirement for immunosuppressive therapy after transplantation (Baniadam et al., 2021).

Furthermore, purified human-induced pluripotent stem cell-derived cardiomyocytes (iPS-CM) were injected directly into the myocardium after MI. There is an improvement after 4 weeks of transplantation. The infarct area was significantly less than in the control group. Through paracrine action, transplanted cells increased angiogenesis and maintained the survival rate of surrounding residential CMs, perhaps leading to functional improvement. Because CMs are a terminal stage of iPS and have limited proliferating ability, no teratomas were observed in any part of the heart (X. Jiang et al., 2020).

The release of exosomes from iPSC-CMs provides a novel type of iPSC-based cardiomyogenesis treatment for heart regeneration as an alternative to direct cell replacement (Ong et al., 2018). Exosomes have antiapoptotic properties, increase angiogenesis, reduce infarct size, and aid in heart recovery. Exosomes obtained from MSCs co-cultured with iPSC-CM boosted prolonged survival and better therapeutic effects in cells after transplantation compared to those derived from iPSC-CMs alone (Yoshida et al., 2018).

In addition, the next study involves the first human trial using clinical-grade hiPSC-CM patches to treat ischemic cardiomyopathy in a patient (Miyagawa et al., 2022). Ischemic cardiomyopathy develops when a patient has a previous myocardial infarction. In the clinical case, no severe arrhythmias or tumors were detected after transplantation. It showed that clinical grade hiPSC-CM patches are non-tumorigenic and non-arrhythmogenic, suggesting that they could be a safe approach to the delivery of cardiac cells (Chow et al., 2017). In the recipient heart, transplanted cardiomyocyte patches have been observed to undergo synchronous contraction/relaxation. After transplantation, the injured myocardium demonstrated time-course healing. Table 6 highlights the current clinical trials carried out using iPSC in cell therapy applications.

**TABLE 6:** Highlights current clinical trials of the use of MSCs in cell therapy applications.

Clinical trial identifier	iPSCs source	Title	Objective	Phase	Status	Trial duration	Country
NCT03883750	Induced pluripotent stem cells	Induced pluripotent stem cells in Niemann Pick Disease (IPSNPABC)	Generate patient-specific induced pluripotent stem cells and then differentiate them into neural cells to study misfolded proteins in the endoplasmic reticulum, their role in the response of untranslated proteins, and possible mechanisms to shuttle misfolded proteins into lysosomes	N/A	Completed	19 June 2018–1 December 2019	Pakistan
NCT03872713	Induced pluripotent stem cells	Establishment of Human Cellular Disease Models for Morquio Disease (IPSMORQUIO)	To prepare a cell culture from patients affected with Morquio disease to identify novel pathways and proteins involved in disease progression that allow for an earlier diagnosis	N/A	Completed	26 October 2018–01 December 2019	Pakistan
NCT03867526	Induced pluripotent stem cells	Establishment of Human Cellular Disease Models for Wilson Disease (IPSWILSON)	Create a human cellular model for addressing hepatic and neurologic forms of the disease	N/A	Completed	19 June 2018–01 December 2019	Pakistan
NCT02923375	Induced pluripotent stem cells	A Study of CYP-001 for the Treatment of Steroid-Resistant Acute Graft *Versus* Host Disease	To assess the safety, tolerability, and efficacy of two CYP-001 infusions in adults with steroid-resistant GvHD.	Phase 1	Completed	01 March 2017–28 August 2018	Australia, United Kingdom
NCT01534624	Induced pluripotent stem cells	Stem Cell Study of Genetics and Drug Addiction	To study genetic and cell differences between people who are addicted to drugs and those who are not	N/A	Completed	07 February 2012–30 July 2014	United States
NCT03372746	Induced pluripotent stem cells	Generation of Induced Pluripotent Stem Cell Lines (iPS) from Skin Fibroblast Cells of Participants with Age-Related Macular Degeneration	Establish a bank of samples that can be changed into other cell types, such as eye cells, to better understand diseases such as AMD, and to test drugs in order to treat various eye diseases	N/A	Completed	23 Ma y 2018–21 March 2019	United State

## 7 Therapeutic approaches using MSCs and iPSCs in COVID-19

The new coronavirus disease 2019 (COVID-19) has drawn the attention of researchers from numerous sectors. The severe acute respiratory syndrome coronavirus 2 (SARS-CoV-2) causes COVID-19, which is a viral acute respiratory disease. COVID-19 mostly harms the lungs and other organs and systems like the heart, immunological system, and so on. Researchers have tried several experiments to develop an appropriate therapeutic option to combat the COVID-19 virus in patients. There are significant concerns about COVID-19 rapid proliferation, although several efforts to create therapeutic platforms have begun, but there is no proper treatment. Cell-based treatment, particularly stem cells, has both therapeutic and preventive potentials.

Stem cells can self-renew and differentiate into multiple types, making them a good option for cell treatment in COVID-19. Stem cell therapy is one of the first treatments to develop for diseases that had not been adequately cured (Du et al., 2020). In this pandemic, stem cell therapies, particularly MSC- and iPSC-related therapies, have demonstrated their therapeutic potential for newly emerging diseases. MSCs have been widely used to study COVID-19-associated diseases, as they release molecules that have potential against the disease. Examples of COVID-19 associated diseases are SARS-CoV-2 pneumonia, COVID-19 pneumonia, acute respiratory distress syndrome (ARDS), and so on. Table 7 summarizes the data of using MSCs in treated patients with COVID-19-associated diseases.

**TABLE 7:** Summary of therapeutic approaches using MSCs in COVID-19 patients.

Sources	COVID-19 associated diseases	Key findings	Key molecules	Study group	Year	References
Mesenchymal stem cell-derived exosomes (MSC-exosomes)	SARS-CoV-2 Pneumonia	Exosomes derived from mesenchymal stem cells (MSC-Exo) have anti-inflammatory and immune modulatory properties, as well as the ability to promote tissue regeneration, suggesting that they are a potential treatment option for SARS-CoV-2 pneumonia. When treating severe pneumonia caused by SARS-CoV-2, it is crucial to suppress the proinflammatory immune response, thereby reducing damage to both alveolar epithelial cells and capillary endothelial cells. MSC-Exo may be able to restore lung cell functionality and restore lung tissue	Keratinocyte growth factor (KGF), vascular endothelial growth factor (VEGF), and hepatocyte growth factor (HGF) help in regenerating damaged tissues	N/A	2020	Akbari and Rezaie, (2020)
Bone marrow-derived mesenchymal stem cells (BM-MSCs)	COVID-19 pneumonia	MSC transplantation led to significant improvements in lung function and symptoms in 2 days. The accumulation of MSCs in the lungs may improve the lung microenvironment, protect alveolar epithelial cells, prevent pulmonary fibrosis, and improve lung function. MSCs were injected intravenously into severe patients with COVID-19 and significantly improved the inflammation situation	Increased levels of IL-10 and VEGF help repair lung damage	COVID-19 patients	2020	Leng et al. (2020)
			The C-reactive protein acts as a biomarker that measures inflammation and host response to cytokine production			
Human umbilical cord mesenchymal stem cell (UC-MSC)	COVID-19 with ARDS	Improved critically ill patients. UC-MSCs were safe and did not cause serious adverse events in patients with moderate and severe COVID-19	Tocilizumab was used to block the IL-6 receptor that helps to improve clinical outcomes in patients with severe COVID-19 patients	COVID-19 patients	2020	Meng et al. (2020)
Exosomes derived from human umbilical cord mesenchymal stem cell-derived exosomes (UC-MSC-Ex)	Inflammation-induced lung damage	There are no side effects for the patients. hUC-MSCs migrate to the damaged area and replace cells. Pulmonary imaging shows an improvement in treated patients. Using MSCs at the early stage of the inflammatory factor storm, the prognosis for COVID-19 may be enhanced	Increase in anti-inflammatory factors such as IL-10	COVID-19 patients	2021	Wei et al. (2021)

### 7.1 Therapeutic approaches using MSCs in COVID-19

Mesenchymal stem cells have the potential to treat SARS-CoV2 pneumonia by secreting exosomes. These cells are commonly considered the most effective regenerative medicine tool, as they can repair damaged tissues and organs. They also release a variety of chemokines, cytokines, growth factors, and extracellular vesicles (T. Zhao et al., 2019). It also helps stimulate regeneration through the production of soluble substances and exosomes to reduce inflammation and promote tissue regeneration (Ullah et al., 2019). MSC transplantation significantly improved the lung function of individuals with 2019-nCov pneumonia in 2 days (Leng et al., 2020). Intravenous infusion of MSCs could minimize immune system overactivation and assist in healing by altering the pulmonary microenvironment after SARS-CoV2 infection.

In the latest study, BM-MSC exosomes were administered to 24 patients aged 18–85 to determine safety and efficacy within 14 days after treatment. There was no adverse reaction after infusion. This study has a high survival rate and cure rate compared to the mortality rate (Sengupta et al., 2020).

Furthermore, human umbilical cord mesenchymal stem cells have the potential to reduce and heal inflammation-induced lung damage caused by COVID-19 (Wei et al., 2021). In patients with COVID-19 who were treated with hUC-MSC, it improved the oxygenation index and decreased IgM levels. Furthermore, the lung imaging of treated patients improved drastically. The infusion of MSCs did not show any allergic response in patients. hUC-MSCs have shown the ability to move into the wounded lung region and differentiate into alveolar cells (Fu et al., 2019). These cells prevent further lung injury and promote regeneration of wounded lung tissues. The severity of lung inflammation was dramatically reduced. Intravenously injected MSCs in patients with COVID-19 were effective and tolerable.

### 7.2 Therapeutic approaches using iPSCs in COVID-19

Induced pluripotent stem cells (iPSCs) are human cells that can self-renew indefinitely and develop into a variety of somatic cells (Sharma et al., 2020). Furthermore, iPSCs are well established and widely discovered by researchers to combat COVID-19. Human iPSCs can be used as an *in vitro* predictive model for SARS-CoV-2 infection and as a screening platform. iPSCs could be one of the best sources for creating patient-specific organs that have fewer complications after implantation. Since the targeted tissues in COVID-19 patients are the lung, it shows a significant impact on the patient’s prognosis (Carsana et al., 2020). Therefore, lung transplantation employing iPSC technology has positive results and does not present immunological rejection (Basiri et al., 2021). SARS-CoV-2 infections have recently been simulated in various organs using organoid and cellular models derived from iPSC, including the heart, brain, liver, intestines, and pancreas. Table 8 summarizes the data of using iPSCs in the study of patients with diseases associated with COVID-19.

**TABLE 8:** Summarizes the therapeutic approaches using iPSCs in COVID-19 associated diseases.

Sources	Diseases	Key findings	Key molecules	Signalling pathway involve	Study group	Year	References
Alveolar organoids	SARS-CoV-2	Lung organoids serve as a pathophysiological model for studying the mechanism of SARS-CoV-2	Remdesivir reduced the viral load of SARS-CoV-2 infection	Down-regulated lipid metabolism upon SARS-CoV-2 infection	Ciliated, club, and alveolar type 2 cells	2021	Pei et al. (2021)
Brain organoids	Neurological complications	Brain organoids as a platform to investigate the susceptibility to SARS-CoV-2 infection of brain cells, a mechanism of virus-induced brain dysfunction	Choroid plexus organoids (CPOs) act as a better model system by expressing SARS-CoV-2 receptors at a high level and exhibit infection and pathogenesis at the cellular and molecular levels	N/A	Human IPSCs from healthy donors and convalescent serum from COVID-19 patients	2020	Jacob et al. (2020)
Retinal organoids	Human eye problem	Study whether ACE2 expression affects human retinal neurons during SARS-CoV-2 infection	ACE2 plays an important role in the regulation of blood pressure and the expression of angiotensin II during inflammation. ACE 2 facilitates entry of SARS-CoV-2 infection	N/A	N/A	2021	Ahmad Mulyadi Lai et al. (2021)
Human IPSC-derived neural stem/progenitor cells (hiPSC-NS/PCs	Central nervous system dysfunction	The viral virulent factor CCN1 has been expressed in cells induced by SARS-CoV-2. CCN1 is involved in viral toxicity. HiPSC-NS/PCs were used as a model to study the SARS-CoV-2 infection-ACE2-CCN1 axis	Compound 34 and γ-secretase inhibitor (GSI) help to reduce the CCN1 level	N/A	N/A	2020	Kase and Okano, (2020)
Human islets and adult hepatocyte and cholangiocyte organoids	Studies on the viral tropism of SARS-CoV-2 and cellular responses to infection	The expression profile and the permissiveness of pancreatic alpha and beta cells to infection by the pseudo-entry virus were confirmed in primary adult human islets. Hepatocytes and cholangiocytes from adult liver organoids were also tested for susceptibility to the SARS-CoV-2 virus	The interaction of cytokine-cytokine receptors, IL-17 signaling, chemokine signaling pathway, TNF signalling pathway, and NF-κB signalling pathway has been overrepresented and upregulated	Chemokine genes including CXCL1, CXCL3, CXCL5, CXCL6, and CCL20 were robustly upregulated, whereas key metabolic markers such as CYP7A1, CYP2A6 and CYP1A2 were significantly downregulated	Cardiomyocytes, dopaminergic neurons, macrophages, microglia, and cortical neurons	2020	(L. Yang et al., 2020)
			Cell metabolism was largely downregulated				
human-induced pluripotent stem cell-derived cardiomyocytes (iPS-CMs)	Cardiac injury	Cardiomyocytes appeared to be less susceptible to infection and cytotoxicity caused by SARS-CoV-2 compared to TMPRSS2+ CaCo-2 cells. Cardiomyocyte infection is typically caused by exposure to a high local virus concentration and for a longer period	Cathepsins L and B, which can also activate the virus, can also activate the S-protein	Activation of interferon pathways is a typical transcriptional response to SARS-CoV-2 infection	Caco-2 cells	2020	Bojkova et al. (2020)
			A protease inhibitor, ALLM, which inhibits cathepsin L and B, reduced the expression of viral spike proteins				
Organoids of human pluripotent stem cell-derived choroid plexus organoids	SARS-CoV-2 Neurotropism	Using an organoid platform, we provide information on the cellular susceptibility, pathogenesis, and treatment strategies associated with SARS-CoV-2 infection of brain cells. In addition, the choroid plexus is suggested to be a potentially important site for disease pathogenesis. CPOs express high levels of SARS-CoV-2 receptors, as the expression level is higher compared to other brain organoids	Monolayer culture includes hiPSC-derived cortical neurons, astrocytes, and microglia, and brain organoids including hiPSC-derived cortical, hippocampal, hypothalamic, and midbrain organoids	Nuclear factor B (NF-B) and mitogen-activated protein kinases (MAPK) are activated by stimulating tumor necrosis factor alpha (TNF-alpha), thus inducing both innate and adaptive immune responses. In other cells, this pathway has been shown to be activated after SARS-CoV-2 infection	N/A	2020	Jacob et al. (2020)
Human-induced pluripotent stem-cell-derived kidney organoids	Kidney fibrosis	Kidney organoids can be successfully infected with SARS-CoV-2 with entry factors such as ACE2 and TMPRSS2. SARS-CoV-2 infected kidney cells induced increased profibrotic signaling, cellular damage, and higher inflammatory responses. This organoid study demonstrates the fundamental pathophysiological effects caused by kidney cell infection	A protease inhibitor (MAT-POS-b3e365b9-1) was used to reduce intracellular viral RNA of SARS-CoV-2	The proinflammatory and fibrosis-driving pathway, such as tumor necrosis factor-alpha (TNFα), transforming growth factor beta (TGFβ), nuclear factor κB (NFκB), and JAK-STAT, shows higher activity in COVID-19 patients	COVID-19 human kidney tissue	2022	Jansen et al. (2022)
Small intestinal epithelial cells derived from human-induced pluripotent stem cells (iPSCs) (iPSC-SIECs)	Gastrointestinal complications	Study whether iPSC-SIECs can be used as a model to investigate gastrointestinal problems. Remdesivir has the potential to reduce gastrointestinal symptoms	N/A	N/A	Remdesivir and a coronavirus fusion inhibitor EK1 inhibit SARS-CoV-2 infection	2022	Yamada et al. (2022)

iPSCs have been used currently by researchers to study and design a protocol to overcome SARS-CoV-2 in patients. COVID-19 causes gastrointestinal symptoms such as diarrhea, nausea, and vomiting, as well as respiratory failure. In this study, human-induced pluripotent stem cell (iPSC)-derived small intestinal epithelial cells (iPSC-SIEC) could be employed as a SARS-CoV-2 infection model (Yamada et al., 2022). SARS-CoV-2 infection was found in absorptive and Paneth cells from iPSC-SIECs. Infection with SARS-CoV-2 also reduced transepithelial electrical resistance (TEER) and increased proinflammatory gene expression. Various intestinal disorders, such as inflammatory bowel diseases, which induce nausea, vomiting, and diarrhea, are more likely to be caused by epithelial barrier damage. COVID-19-induced gastrointestinal symptoms could be caused by barrier injury of the intestinal mucosa caused by viral infection. In SIECs, infection with SARS-CoV-2 increased the expression of proinflammatory genes. Increased cytokine levels have often been observed in patients with severe COVID-19 (L. Zhou et al., 2020, p. 2). The gastrointestinal tract was also involved in broad systemic inflammation in deceased COVID-19 individuals.

Remdesivir also decreased SARS-CoV-2 infection and reversed SARS-CoV-2-induced barrier degradation and inflammatory responses. The disruption of the mucosal barrier and inflammatory reactions after SARS-CoV-2 infection could have caused these gut injuries. Remdesivir suppresses the cytokine storm in patients with COVID-19, implying that remdesivir reduces patients’ gastrointestinal problems (Yamada et al., 2022).

Additionally, SARS-CoV-2 infection affects other organs than the lungs. Neurological tissues and eyes have been observed to be affected in some patients with COVID-19. Different studies showed that ACE2 expression was found in the human cornea and conjunctiva (L. Zhou et al., 2020). Furthermore, SARS-CoV-2 was discovered in postmortem retinal samples from COVID-19 patients (Casagrande et al., 2020). This study was to check whether the expression of ACE2 in human retinal neurons has a function during SARS-CoV-2 infection. Thus, hiPSC retinal organoids and monolayer cultures from dissociated retinal organoids were created (Ahmad Mulyadi Lai et al., 2021).

SARS-CoV-2 pseudovirus was used, which is based on the lentiviral system and contains the S protein on the surface of the capsid. Dissociated retinal organoids in monolayer culture were sensitive to low MOI of the SARS-CoV-2 pseudovirus. In retinal organoids, ACE2 acts as a virus entry channel. RNA sequence analysis and bioinformatic analysis were performed to identify genes expressed differently in infected organoids and monolayer cultures (Ahmad Mulyadi Lai et al., 2021). The RNA sequence shows that the retinal organoids and monolayer cultures regulated epithelial cell apoptosis and the inflammatory response. Inflammatory responses caused by SARS-CoV-2 are the leading cause of disease development in COVID-19 patients (García, 2020). The genes in the inflammatory response, such as F3 and SEMA7A, appear higher in SARS-CoV-2 pseudovirus infected retinal organoids and monolayer cultures than in uninfected controls.

This can be concluded that both retinal organoids and monolayer cultures express ACE2 and are likely to be infected by the SARS-CoV-2 pseudovirus. This shows that human iPSC-derived retinal organoid and monolayer cultures are the best disease platform to study viral entry.

## 8 Summary

In summary, after decades of research, stem cell therapy is proving to be a tremendous game changer in medicine. The potential of stem cells develops with each experiment, yet there are still numerous challenges to overcome. The use of MSCs for therapeutic purposes is significant because they are easily available from different sources, they can produce in large quantities, and they have a simple isolation procedure. Currently, MSCs are one of the most vital cells widely utilized in clinical and preclinical trials due to their valuable advantages. The challenges in MSCs involve using autologous and allogeneic MSCs for clinical application. Allogeneic MSCs appear to be used more frequently in preclinical studies and clinical trials. Transplantation of autologous MSCs has some limitations. The first problem is the high cost of preparing cells for one recipient alone. It is also challenging to obtain a clinical dose of MSCs from some patients. MSCs isolated from older donors have decreased proliferation, differentiation, and regenerative potential, resulting in ineffective therapies (Marędziak et al., 2016). On the contrary, it is evident that the use of allogeneic *versus* autologous MSCs for cell therapy has clear advantages. To solve this problem, allogeneic MSCs from healthy young donors are the best option. Furthermore, autologous MSCs expand at a slow rate, making this therapeutic approach difficult to use for early treatment of acute diseases. The benefit of allogeneic MSCs is that once obtained, they can be frozen and stored, then rapidly thawed and administered immediately to the patient (García-Bernal et al., 2021).

On the other hand, iPSCs are crucial, as MSCs are limited in number and differentiation potential. Human-induced pluripotent stem cells (hiPSCs) are a promising alternative to embryonic cells, eliminating ethical concerns. Clinical trials using iPSCs are less expensive and reduce immunorejection rates. Despite the advances made so far, more intense research is required on human iPSCs to understand the basic biology of pluripotency and cellular differentiation to address all challenges related to therapeutic applications. To generate and expand iPS cells, it requires a high cost, including the necessary experiments to validate the safety and pluripotency. It also requires a longer period of time for production. To overcome this issue, allogeneic therapies, which are more economical and widely available for clinical use. Regarding clinical trials involving MSCs and iPSCs, more studies have been recorded using MSCs compared to iPSCs from 2018 to 2021. This is because MSCs are well established and commonly used in therapeutic applications, whereas iPSCs are relatively new and need to be improved to obtain clinical-grade human iPSCs for safe cell treatment. We project that hiPSCs would further dominate stem cell-based therapy due to their availability if most of their limitations can be addressed.
